# Is Carbon Heteroatom Doping the Key to Active and Stable Carbon Supported Cobalt Fischer–Tropsch Catalysts?

**DOI:** 10.1021/acscatal.4c08092

**Published:** 2025-04-09

**Authors:** Felix Herold, Dominic de Oliveira, Göran Baade, Jens Friedland, Robert Güttel, Michael Claeys, Magnus Rønning

**Affiliations:** † Department of Chemical Engineering, Norwegian University of Science and Technology, Trondheim 7491, Norway; ‡ Institute for Power-to-X Technologies, Friedrich-Alexander-Universität Erlangen-Nürnberg, 90762 Fürth, Germany; § Department of Chemical Engineering, Catalysis Institute, University of Cape Town, Rondebosch, Cape Town 7701, South Africa; ∥ Institute of Chemical Engineering, Ulm University, 89081 Ulm, Germany

**Keywords:** Fischer–Tropsch synthesis, carbon heteroatom doping, catalyst deactivation, X-ray absorption spectroscopy, X-ray diffraction, magnetometry

## Abstract

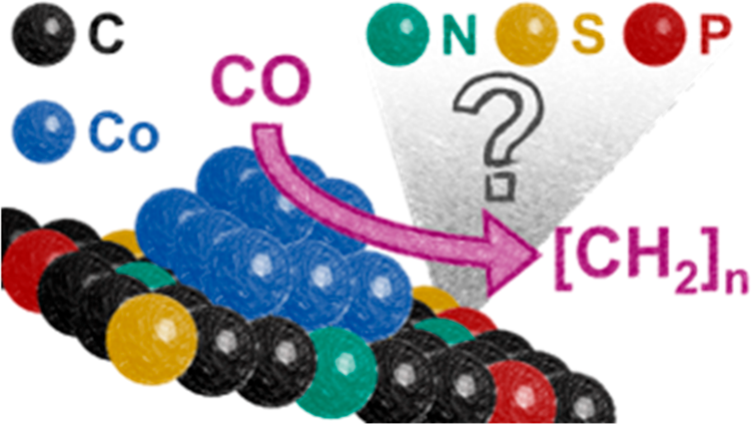

Carbon supports are an interesting alternative to established oxidic catalyst supports for Co-based Fischer–Tropsch synthesis (FTS) catalysts as they allow high Co reducibility and do not suffer from the formation of Co/support compounds. To optimize Co-based carbon-supported FTS catalysts, significant research has focused on doping carbon supports with heteroatoms, aiming to enhance both catalytic activity and stability. While improvements in FTS performance have been reported for N-doped carbon supports, the exact effects of heteroatom doping are still poorly understood, largely due to difficulties in directly comparing Co FTS catalysts supported on doped versus nondoped carbon materials. In this study, we synthesized a series of highly comparable N-, S-, and P-doped carbon nanofiber (CNF) model supports, which were combined with size-controlled, colloidal Co nanoparticles to create well-defined model FTS catalysts. Comprehensive characterization of these catalysts using in situ X-ray absorption spectroscopy (XAS), in situ X-ray diffraction (XRD), and in situ magnetometry revealed that the presence of dopants significantly altered the structure and properties of the catalytically active Co^0^ phase, affecting Co coordination numbers, crystal phase composition, and magnetic behavior. Challenging optimistic literature reports, our findings demonstrate that all the studied heteroatoms negatively impact either FTS activity or catalyst stability. Co on N-doped CNFs experienced rapid deactivation due to increased sintering as well as Co phase transformations, which were not observed for Co on nondoped CNFs. Co on S-doped CNF suffered from instability of carbon-bound S species in a hydrogen atmosphere, contributing to low FTS performance by S-poisoning. Finally, Co on P-doped CNFs displayed strong metal–support interactions that improved sintering stability, but FTS activity was hampered by low Co reducibility and the loss of active Co^0^ due to a complex sequence of cobalt phosphide formation and its subsequent decomposition into phosphorus oxides and cobalt oxide species under FTS conditions.

## 1 Introduction

In pursuit of sustainable aviation fuels and chemical building blocks, the Fischer–Tropsch synthesis (FTS) is a process of high interest that can produce hydrocarbons from a mixture of CO and H_2_ (syngas), sourced by combining renewable carbon sources with green hydrogen.^1,2^ A widely studied catalyst system for FTS are cobalt nanoparticles (Co NPs) dispersed on a suitable catalyst support, that stabilizes the active metal phase (Co^0^) and provides large surface areas and anchoring points for cobalt nanoparticles.^3^ Porous oxide catalyst supports such as γ-Al_2_O_3_, SiO_2_, and TiO_2_ satisfy the latter requirement well but do not necessarily stabilize the active Co^0^ phase as they suffer from low Co reducibility^4^ and from the formation of nonregenerable metal/support compounds (e.g., Co_*x*_Al_*y*_O_*z*_).^4−7^ Potential alternatives are carbon supports, which facilitate high Co reducibility and do not form metal–support compounds.^8^ However, carbon supports often fail to provide sufficient anchoring for Co NPs, promoting catalyst deactivation by Co sintering.

A major advantage of carbon supports is their tunability, with the introduction of heteroatoms (e.g., N, S, P) into the carbon lattice being a common approach to optimize metal–carbon interactions in carbon-supported catalysts.^9,10^ In the context of carbon-supported Co-based FTS catalysts, N doping received major attention, with numerous reports of Co supported on N-functionalized carbon nanotubes,^11−14^ carbon spheres,^15−17^ mesoporous carbon,^18,19^ graphene,^20,21^ carbon aerogel,^22^ polydopamine-derived carbon,^16,23^ metal organic framework (MOF)-derived carbon,^24−28^ or even N-doped carbon as promotor for SiO_2_-supported Co catalysts.^29^ Indeed, literature studies report higher Co reducibility^13,19,20^ as well as superior FTS activity^13,16,17,19,20^ and stability^13,16,17^ of Co on N-doped carbon supports as compared to nondoped carbons.

Despite these efforts, the exact effects of carbon heteroatom doping on Co-based FTS catalysts are still poorly understood. This is primarily an issue of lacking comparability, as apple-to-apple comparisons of Co-based FTS catalysts supported on doped and nondoped carbon are very challenging to realize. Regarding the carbon supports, it is highly demanding to synthesize carbon materials that only differ in the presence or absence of heteroatoms but not in other factors of influence. Commonly, introduction of heteroatoms such N, S, or P alters simultaneously the texture and nanostructure of the carbon material, rendering the isolation of the effects of heteroatom doping challenging as multiple important factors of influence are changed at once.^30^

Regarding the active Co phase, comparability of Co-based FTS catalysts is complicated by the presence of well-known nonclassical particle size effects.^5,31,32^ Co NPs below a threshold of 6–8 nm show a significant decline in intrinsic FTS activity and C_5+_ selectivity, thus preventing any meaningful comparison of FTS performance of catalysts with diverging Co NP size distributions. For the preparation of Co-based FTS catalysts supported on heteroatom-doped carbon, metal impregnation(deposition)/calcination procedures dominate literature reports,^11,12,14,16,17,19−21^ during which Co NP formation takes place directly on the support surface. As heteroatom-doped and nondoped carbon surfaces display vastly different properties (e.g., polarity, amount/type/reactivity of adsorption sites), the formation of noncomparable Co NP size distributions is hardly preventable,^12,16,19^ rendering the isolation of the effects of carbon heteroatom doping on FTS performance all but impossible.

In this study, we aimed to isolate the effects of the heteroatom dopants N, S, and P on the activity and stability of carbon-supported Co FTS catalysts. In this context, a comparable series of doped and nondoped carbon nanofiber (CNF) supports was prepared by gasification-assisted heteroatom doping (GAHD).^30^ GAHD is a specifically developed postsynthesis heteroatom doping procedure that enables the introduction of N, S, and P into carbon lattices while otherwise retaining the structural features of the parental carbon. A series of comparable nondoped and N-, S-, and P-doped CNF supports of similar heteroatom loading was combined with size-defined, colloidal Co NPs to create highly defined model catalysts. Comprehensive (in situ) characterization of the catalyst models was combined with FTS performance data, aiming to isolate the effects of N, S, and P doping of the CNF supports on catalyst activity and deactivation.

## 2 Materials and Methods

### 2.1 Materials

All gases were purchased from Linde AG. Nitric acid (65 wt %), ethylene diamine, carbon disulfide, trimethyl phosphite, oleic acid (90%, technical grade), 1,2-dichlorobenzene (99%, anhydrous), and dicobalt octacarbonyl (90%) were purchased from Sigma-Aldrich. *n*-Hexane and toluene were acquired from Merck, while isopropanol, acetone, and 1-octadecene (90%) were purchased from Fisher Scientific. Cyclohexane (>99.5%) was acquired from VWR. CoO and Co_3_O_4_ powder were acquired from Alfa Aesar. It should be noted that all volumetric flow rates reported in the following are specified for standard conditions (STP).

### 2.2 Carbon Nanofiber Synthesis

The synthesis of platelet-type carbon nanofibers was executed according to a procedure of Yu et al.^33^ In a vertical tube furnace equipped with a quartz reactor (40 mm internal diameter) and a ceramic frit, a Fe_3_O_4_ catalyst was exposed to 100 mL min^–1^ of 25 vol % H_2_ in Ar at 600 °C for 6 h. The system was subsequently flushed with 100 mL of pure Ar for 30 min, before introducing the reaction mixture of 50 mL min^–1^ CO and 12.5 mL min^–1^ H_2_. After performing chemical vapor deposition (CVD) for 48 h at 600 °C, the reactor was flushed with Ar and allowed to cool. To remove the Fe growth catalyst, the CNF was treated with HNO_3_, by stirring the CNF in 65 wt % HNO_3_ (HNO_3_/CNF weight ratio of 15) for 24 h at 120 °C. The CNF was subsequently filtrated and washed with deionized water until the effluent showed neutral pH. This HNO_3_ washing procedure was repeated three times, yielding around 20 g of purified CNF after drying at 100 °C in air. It should be noted that all catalyst supports discussed in this study originate from a single batch of purified CNF.

### 2.3 High-Temperature Hydrogen Treatment

To synthesize a CNF reference support with a surface mostly terminated by H, purified CNF was subjected to a high-temperature treatment in H_2_. In a vertical tube furnace, 2 g of purified CNF was heated at a rate of 10 °C min^–1^ to 950 °C in a flow of 100 mL min^–1^ H_2_. After keeping the sample at 950 °C for 30 min, the setup was flushed with Ar and allowed to cool. The resulting catalyst support was designated “CNF-H”.

### 2.4 Heteroatom Doping

Heteroatom doping was carried out by gasification-assisted heteroatom doping,^30^ a procedure relying on the introduction of carbon surface defects by a gasification agent and subsequent saturation of these defects by a simultaneously present heteroatom source. The procedure relied on a vertical tube furnace holding a quartz reactor equipped with a ceramic frit and an internal type K thermoelement as reactor. N, P, and S sources were supplied as vapors, by sparging a carrier gas flow through the liquid heteroatom sources (ethylene diamine, carbon disulfide, trimethyl phosphite) in a saturator system upstream of the reactor. H_2_O, CO_2_, and H_2_ were employed as carbon gasification agents and were supplied either as vapor via the saturator system (H_2_O) or via mass flow controllers (CO_2_, H_2_). Gases were supplied using a gas distribution system that enabled a flexible gas routing either through the saturator or to a bypass before entering the reactor.

#### 2.4.1 N Doping

350 mg of purified CNF was heated at a rate of 10 °C min^–1^ to 875 °C in a flow of 250 mL min^–1^ of N_2_. After reaching 875 °C, the N_2_ flow was redirected through the saturator, which contained a room temperature mixture of 1:1.5 mol mol^–1^ of ethylene diamine and water. After feeding the ethylene diamine/H_2_O vapors at 875 °C for 1.5 h, the N_2_ flow is redirected to bypass the saturator, and the system is allowed to cool. 310 mg of N-doped carbon nanofibers designated “CNF-N” was obtained. Before Co loading, several batches of CNF-N were mixed after ensuring via X-ray photoelectron spectroscopy (XPS) that the desired level of N loading was achieved.

#### 2.4.2 S Doping

350 mg of purified CNF was heated at a rate of 10 °C min^–1^ to 825 °C in a total flow of 50 mL min^–1^ of 20 vol % CO_2_ in N_2_. Reaching 825 °C, a flow of 2 mL min^–1^ N_2_ was passed through the cooled (−20 °C, cooled by a NaCl/ice bath) saturator filled with carbon disulfide and combined with a flow of 48 mL min^–1^ 20 vol % CO_2_ in N_2_ before entering the reactor. After feeding the reaction mixture for 7.5 min at 825 °C, the reactor was flushed with a flow of pure N_2_ and allowed to cool. 300 mg of S-doped carbon nanofibers designated “CNF-S” was collected. Several batches of CNF-S were mixed after ensuring via XPS that the desired level of S loading was achieved. Before Co loading, the combined batches were subjected to a heat treatment in a flow of 50 mL min^–1^ H_2_, employing a heating rate of 10 °C min^–1^ to 450 °C, which was held for 1 h. The heat treatment in hydrogen did not change the S loading, as confirmed by XPS.

#### 2.4.3 P Doping

350 mg of purified CNF was heated to 825 °C at a rate of 10 °C min^–1^ in a flow of 50 mL min^–1^ H_2_. Reaching 825 °C, the gas flow was redirected through a saturator filled with trimethyl phosphite. After 1.5 h, the saturator was bypassed, the reactor was flushed with a flow of N_2_, and the system was allowed to cool. After reaching room temperature, a flow of 100 mL min^–1^ of synthetic air was passed through the reactor overnight, in order to oxidize any traces of white phosphorus that may have formed during the doping procedure. 310 mg of P-doped carbon nanofibers designated as “CNF-P” was obtained. Before Co loading, several batches of CNF-P were mixed after ensuring via XPS that the desired level of P loading was achieved.

### 2.5 Co Nanoparticle Synthesis and Loading

Synthesis and loading of colloidal Co nanoparticles was executed according to procedures by van Deelen et al.^34,35^ In a 100 mL three-neck flask equipped with a cooler, two septa, a magnetic stirrer, and a connection to a Schlenk line, 73.5 μL of oleic acid were degassed in vacuum at 100 °C while stirring at 150 rpm. The system was subsequently flushed with N_2_, 7.5 mL dry 1,2-dichlorobenzene was added, and it was heated to 174 °C. Meanwhile, in a glovebox, 270 mg dicobalt octacarbonyl was dissolved in 1.5 mL 1,2-dichlorobenzene and rapidly injected into the hot reaction mixture while stirring at 750 rpm. After 20 min at 174 °C, the Co nanoparticle (Co NP) dispersion was quenched in a water bath, one septum was removed, and the N_2_ flow from the Schlenk line was stopped to slowly expose the Co NPs to air for 1 h while stirring at 650 rpm. Subsequently, the Co NP dispersion was transferred to a 50 mL centrifuge tube, and isopropanol was added until the total volume was 45 mL. The Co NP dispersion was centrifuged for 20 min at 2200*g*, the supernatant was decanted, the Co NPs were redispersed in 1 mL of *n*-hexane and isopropanol was added until the total volume reached 40 mL. After repeating this washing cycle three times, the Co NPs were redispersed in 2 mL of toluene and stored in a glass vial. After a thorough quality control by scanning transmission electron microscopy (STEM) imaging, several batches of Co NP dispersion were mixed, so that every catalyst discussed in this study was loaded with the same charge of Co NPs consisting of multiple mixed individual Co NP batches.

Co NP loading was carried out by wet impregnation. In a 100 mL three-neck flask equipped with a condenser, two septa, and a magnetic stirrer, 750 mg CNF was suspended in 15 mL 1-octadecene by stirring at 400 rpm for 15 min. 2.4 mL of Co NP dispersion in toluene was added under continuous stirring at 400 rpm, and the suspension was degassed under vacuum at 100 °C for 30 min. Subsequently, the system was flushed with N_2_, heated to 200 °C for 30 min before being allowed to cool. The dispersion was transferred to a 50 mL centrifugation tube, 20 mL acetone was added, and it was centrifuged at 2500*g* for 5 min. After decanting the supernatant, the catalyst was redispersed in 2 mL of *n*-hexane, 6 mL of acetone was added, and the suspension was centrifuged again. This washing cycle was repeated 6 times, before the Co/CNF catalysts were dried overnight at 60 °C in vacuum.

### 2.6 Ex Situ Characterization

Nitrogen physisorption was executed utilizing a Micromeritics Tristar 3020 analyzer at −196 °C after sample degassing at 200 °C and 0.01 Torr overnight. The BET method was used to determine specific surface areas. Raman spectroscopy was performed using a HeNe laser (λ = 633 nm) on a Horiba Jobin Yvon LabRAM HR800 Raman spectrometer. At least 5 spectra of different locations were evaluated with a curve-fitting procedure proposed by Mallet-Ladeira et al.^36^ for each sample. The Co loading of Co/CNF catalysts was analyzed by microwave digestion in *aqua regia* (Berghof SpeedWave XPERT) and subsequent analysis by microwave plasma excited atomic emission spectroscopy (Agilent 4210, MP-AES). An Altamira Benchcat analyzer was utilized for temperature-programmed reduction (TPR), by heating ∼25 mg Co/CNF in a flow of 20 mL min^–1^ 7 vol % H_2_ in Ar at a rate of 5 °C min^–1^ to 800 °C while monitoring the off-gas H_2_ concentration with a thermal conductivity detector (TCD). Scanning transmission electron microscopy (STEM) and energy-dispersive X-ray spectroscopy (EDS) were performed on a Hitachi SU9000 electron microscope operating at an accelerating voltage of 30 kV. EDS mapping was performed using an Oxford Ultim Extreme 100 mm^2^ detector and a pixel dwell time of 400 μs. EDS line scans were conducted with a dwell time of 5 s per point. STEM sample preparation included dispersion in *n*-hexane by ultrasonication followed by drop casting on holey carbon-coated copper grids. Spent catalyst samples were washed thoroughly with *n*-hexane before redispersion and drop casting on sample grids. Co particle size distributions were determined using ImageJ by manually counting 300–500 particles. Average diameters of metallic Co nanoparticles were determined by correcting for a 3 nm surface oxide layer, using the relation d(Co) = 0.75 d(CoO_*x*_) as proposed by Eschemann et al.^37^ For X-ray photoelectron spectroscopy (XPS), a Kratos Analytical Axis Ultra DLD spectrometer was employed, using monochromatic Al Kα irradiation (1486.6 eV) operating the anode at 10 kV with an aperture of 700 × 300 μm. High-resolution spectra were recorded with a pass energy of 20 eV, while survey scans were measured at a pass energy of 160 eV. The energy axis was calibrated by fixing the C 1s contribution of sp^2^ carbon (“graphite”) at 284.6 eV. Before curve fitting with pseudo-Voigt profiles, Shirley background subtraction was executed (detailed fitting procedures are included in the Supporting Information).

### 2.7 In Situ Characterization

Combined in situ X-ray absorption spectroscopy (XAS) and X-ray powder diffraction (XRD) was performed at beamline BM31 of the Swiss-Norwegian beamlines (SNBL) at the European Synchrotron Radiation Facility (ESRF) in Grenoble, France. To this end, tubular quartz capillary reactors (1.5 mm internal diameter, 0.02 mm wall thickness) were loaded with 5–10 mg Co/CNF to reach a bed length of 1 cm and fixed with quartz wool plugs at each end. The reactor was placed in a specifically developed resistance heater^38^ to control the temperature and connected to a gas distribution system. Online off-gas analysis was performed utilizing mass spectrometry (Pfeiffer Omnistar). XRD was performed with a Pilatus3 2 M detector (Dectris) at a wavelength of λ = 0.24486 Å. A NIST 660a LaB_6_ reference was used to account for instrumental broadening and to execute wavelength calibration as well as detector distance corrections. XAS was performed at the Co K edge (7709 eV) in transmission mode. Reduction experiments were conducted in a flow of 0.5 mL min^–1^ of 25 vol % H_2_ in He, whereas the flow rate was chosen to resemble the space velocity used for catalyst reduction before the FTS experiments. Catalyst reduction was conducted by heating from 50 to 350 °C at 3 °C min^–1^ and subsequently holding 350 °C for 1–6 h until no further changes in the collected XANES profiles could be observed. During the reduction experiment, XANES profiles and XRD patterns were recorded, while EXAFS and XRD data was collected at 50 °C before and after the reduction, without any intermediate exposure to air. A Co^0^ hcp foil as well as CoO and Co_3_O_4_ powder served as standards, for which EXAFS data was measured ex situ in transmission mode. In addition, ex situ EXAFS data was measured for the Co NP dispersion in toluene. Ex situ XRD reference data was collected for the plain supports CNF-H, CNF-N, CNF-S, and CNF-P.

XANES and EXAFS data was handled employing the Athena and Artemis components of the Demeter software.^39^ XANES profiles were normalized and subjected to linear combination fitting (LCF) in the range from −20 eV to +40 eV relative to the Co K-edge, with CoO and Co_3_O_4_ powder as well as Co/CNF-H after reduction (for Co^0^) serving as standards. EXAFS fitting was conducted for the reduced catalyst samples by first-shell fitting of *k*^2^ weighted data in the *R* space in the range of 1 < *R* < 3 Å. The number of floating parameters used in the evaluation procedure satisfied the Nyquist criterion.^40^

TOPAS 5.0 (Bruker) was used for Rietveld refinement of in situ XRD patterns. Employing a NIST 660a LaB_6_ reference, emission profiles as well as instrument details were determined. Ex situ measurements of the plain supports were used to approximate the support contribution of XRD patterns by a series of Split Pearson VII profiles, which were subsequently used in the refinement of all further XRD measurements. During refinement of Co/CNF catalyst samples, only scaling of the support pattern was allowed. The XRD patterns of the Co/CNF catalyst samples were refined using database structures for Co hcp (COD-9008492), Co fcc (PDF-00-015-0806), CoO (COD-1541642), as well as Co_3_O_4_ (COD-9005896). Signal broadening due to crystallite size effects was described by Lorentzian functions; strain broadening was excluded. Further refined was a Chebyshev background of fifth order, sample displacement, zero error, and cylindrical 2θ correction. In accordance with other literature procedures,^34^ due to the presence of a cobalt hcp–fcc intergrowth phase, a sensible fit of the reduced catalysts was only obtainable by refinement of the preferred orientation for the Co hcp (100) and (001) directions using the March–Dollase model.

In situ magnetometry was executed using a specifically developed device established at the University of Cape Town, South Africa,^32,41^ employing a tubular fixed bed reactor placed between the poles of an electromagnet, allowing the exposure of catalyst samples to strong external magnetic fields of −2.0 to +2.0 T. The setup allows the examination of phase transitions in ferromagnetic materials such as Fe, Ni, and Co in various atmospheres at elevated temperatures and pressures (e.g., 220 °C and 20 bar). Considering Co, only metallic Co displays ferromagnetism and an appreciable magnetic susceptibility, while cobalt oxides are antiferromagnetic with Néel temperatures well below room temperature, and ferromagnetic cobalt carbides exhibit very low magnetic susceptibilities.^42−44^ In consequence, magnetization of Co/CNF in high external fields of 2 T is very sensitive to the amount of Co^0^ present in the reactor, with catalyst reduction being evident as increasing magnetization while any phase transformation of Co^0^ (e.g., to Co_*x*_O_*y*_, Co_*x*_C_*y*_, Co_*x*_N_*y*_, Co_*x*_S_*y*_, Co_*x*_P_*y*_) results in a decrease of magnetization. For magnetic characterization of the catalyst samples, the saturation magnetization *M*_S_ (average of the magnetization at +2.0 and −2.0 T) as well as the remanent magnetization *M*_R_ (magnetization in absence of an external field after saturation) served as principal parameters. The remanent magnetization is sensitive to the Co particle size as small Co particles below a critical diameter (*D*_c_) display superparamagnetism, meaning that they do not show remanent magnetization.^43,45^ The critical diameter depends on the Co crystal structure, and is still subject to discussion. Bean and Livingston estimated a *D*_c_ of 8 nm for Co hcp and 28 nm for Co fcc at room temperature,^46^ while other studies assume that the critical diameter (disregarding the Co phase) is situated in a range between 15 and 20 nm.^41,45,47^ The ratio of remanent magnetization (*M*_R_) and saturation magnetization (*M*_S_) (γ, with γ = 2 *M*_R_·*M*_S_^–1^), as a measure for the fraction of Co particles exceeding the critical diameter, may serve as a semiquantitative indicator for Co particle size, that allows to follow Co particle growth in situ.^41,45^ In situ magnetometry was used to monitor catalyst reduction as well as FTS, with experimental conditions resembling those used for FTS testing. Saturation magnetization at +2.0 and −2.0 T and remanent magnetization (at 0 T, after prior saturation) were monitored permanently, while full magnetic hysteresis measurements were conducted each at the start and end of catalyst reduction and FTS. 250 mg Co/CNF catalyst was diluted in a 1:4 g g^–1^ ratio by 1 g SiC and placed between two glass-wool plugs in the tubular fixed bed reactor of the magnetometer. A PT100 resistance temperature detector probe, placed within the catalyst bed, measured the reactor temperature, which was controlled by infrared heating. Catalyst reduction was carried out at 350 °C for 8 h in a flow of 12.5 mL of 25 vol % H_2_ in Ar, utilizing a heating rate of 1 °C min^–1^. After reduction, the reactor was cooled in Ar to 190 °C, using a flow of Ar to increase the pressure to 20 bar. Subsequently, syngas (H_2_/CO ratio 2.1, 10 vol % Ar as internal standard) was introduced to replace the initial Ar atmosphere at a flow rate of 6.25 mL min^–1^. After complete replacement of Ar, the reactor was heated at 0.1 °C min^–1^ to 220 °C and kept at that temperature for 24 h.

### 2.8 Fischer–Tropsch Synthesis

A stainless-steel tubular reactor of 4 mm internal diameter was employed for FTS activity measurements. To this end, 100–250 mg Co/CNF was diluted in a 1:4 g g^–1^ ratio by 400–800 mg of SiC and fixed between two glass wool plugs, with a type K thermocouple placed within the catalyst bed controlling the reactor temperature. Catalyst reduction was carried out in situ at atmospheric pressure at 350 °C (heating rate 1 °C min^–1^) for 8 h in a flow of 10 mL min^–1^ of 25 vol % H_2_ in Ar. Subsequently, the reactor was cooled to 190 °C in a flow of 7.5 mL min^–1^ Ar and pressurized to 20 bar in an identical flow of Ar. After reaching 20 bar, syngas was introduced at a total flow rate of 5 mL min^–1^, with a H_2_/CO ratio of 2.1 and 10 vol % Ar as internal standard. After complete replacement of Ar with syngas, the reactor was heated from 190 to 220 °C using a heating rate of 0.1 °C min^–1^, and the temperature was kept constant for 80 h. After the end of each reaction run, spent catalysts were passivated at room temperature and atmospheric pressure in a flow of 10 mL min^–1^ 1 vol % O_2_ in Ar. Online gas chromatography was used for the quantification of gaseous reaction products. To this end, a GC–MS (Agilent GC7890B-MSD5977A) equipped with a flame ionization detector (FID) for the detection and quantification of C_2_–C_5_ hydrocarbons and a TCD for analysis of CH_4_, CO, H_2_, and Ar was employed. Selectivities for C_1_–C_4_ in terms of %(C) were determined by *S*_C_1__–_C_4__ = *V̇*_Cn_·*n*·(*V̇*_CO,in_ – *V̇*_CO,out_)^−1^, while the C_5+_ selectivity was calculated by *S*_C_5+__ = 1 – *S*_C_1__–_C_4__. Higher hydrocarbons collected in the cold trap (4 °C) were analyzed by offline GC (Trace 1300 by Thermo Scientific) equipped with an FID and a simulated distillation column (Agilent DB-2887). Before injection, 0.1 mL of the liquid sample was diluted by 1 mL of cyclohexane.

## 3 Results and Discussion

### 3.1 Heteroatom-Doped Carbon Nanofiber Supports

**Figure 1:**
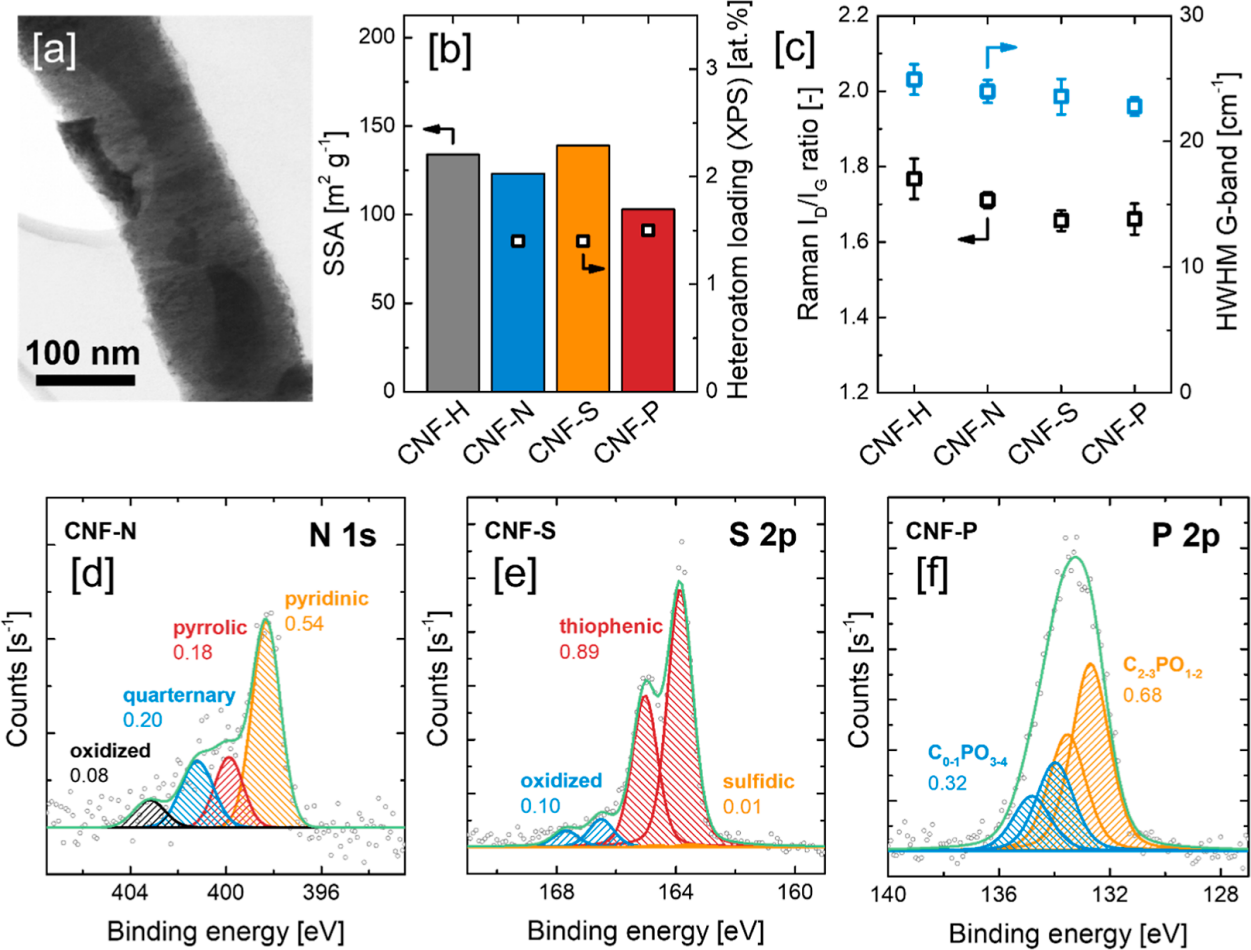
(a) Brightfield STEM image of CNF-H. (b) Specific surface area (BET) and heteroatom loading of the CNF supports. (c) Raman *I*_D_/*I*_G_ ratio and the half-width at half-maximum (HWHM) of the Raman G-band of the CNF supports. Deconvolution of the high-resolution XPS. (d) N 1s contribution of CNF-N, (e) S 2p contribution of CNF-S, and (f) P 2p contribution of CNF-P.

Platelet-type carbon nanofibers were synthesized by chemical vapor deposition, purified, and subjected to postsynthesis heteroatom doping (Figure 1a). For this purpose, gasification assisted heteroatom doping (GAHD) was employed, a procedure specifically developed to dope N, S, and P into carbon surfaces with otherwise minimal impact on the properties of the parent carbon (e.g., texture, nanostructure). GAHD relies on gasification agents (H_2_O, CO_2_, or H_2_) to introduce defects to the carbon surface, while a simultaneously present heteroatom source saturates these newly formed reactive sites, resulting in a net exchange of carbon surface atoms with heteroatoms. To ensure minimal impact of the heteroatom doping procedure on the texture and nanostructure of the parental carbon, the reaction conditions (e.g., temperature, duration, ratio of gasification agent to heteroatom source etc.) of GAHD have to be carefully optimized, which has been done in previous work (see ref (30), which also includes a discussion on advantages and limits of GAHD). Beyond N-, S-, and P-doped CNF, a nondoped reference support was prepared by high-temperature treatment in H_2_, aiming to remove all heteroatoms from the carbon surface.

Employing GAHD, CNF’s were doped with N, S, and P, introducing similar heteroatom surface concentrations between 1.4 and 1.5 at. % (Figure 1b, Table S1; for further information, see ref (30)). N_2_ physisorption experiments revealed-type IVa isotherms without any plateau in N_2_ uptake at *p*/*p*_0_ > 0.9, typical for the micro-, meso-, and macropores that are formed by the interparticle spaces of the otherwise nonporous CNF (Figure S1a). In terms of general shape, the N_2_ isotherms of the doped and nondoped supports were nearly identical, with slight differences in the N_2_ uptake at *p*/*p*_0_ < 0.05, which are probably related to differences in surface roughness (Figure S1b). Specific surface areas were found to be comparable, ranging from 103 m^2^ g^–1^ for CNF-P to 139 m^2^ g^–1^ for CNF-S (Figure 1b). XRD showed the sharp reflections typical for a graphitic carbon material, whereas the diffractograms of the doped and nondoped CNF supports did not show any significant differences (Figure S2). Raman spectroscopy yielded high *I*_D_/*I*_G_ ratios around 1.7, pronounced D′ band (1620 cm^–1^), and clear second-order 2D band (∼2660 cm^–1^) contributions, confirming the graphitic nanostructure with comparatively low in-plane crystallite size and high concentration of carbon edge sites that is expected for platelet-type CNF (Figures 1c and S3). Postsynthesis heteroatom doping only caused minor changes in the carbon nanostructure, as the narrow spread of Raman *I*_D_/*I*_G_ ratio (between 1.77 ± 0.05 for CNF-H and 1.66 ± 0.03 for CNF-S) and half-width at half-maximum (HWHM) of the G band indicate (between 24.9 ± 1.2 cm^–1^ for CNF-H and 22.8 ± 0.7 cm^–1^ for CNF-P).

**Figure 2:**
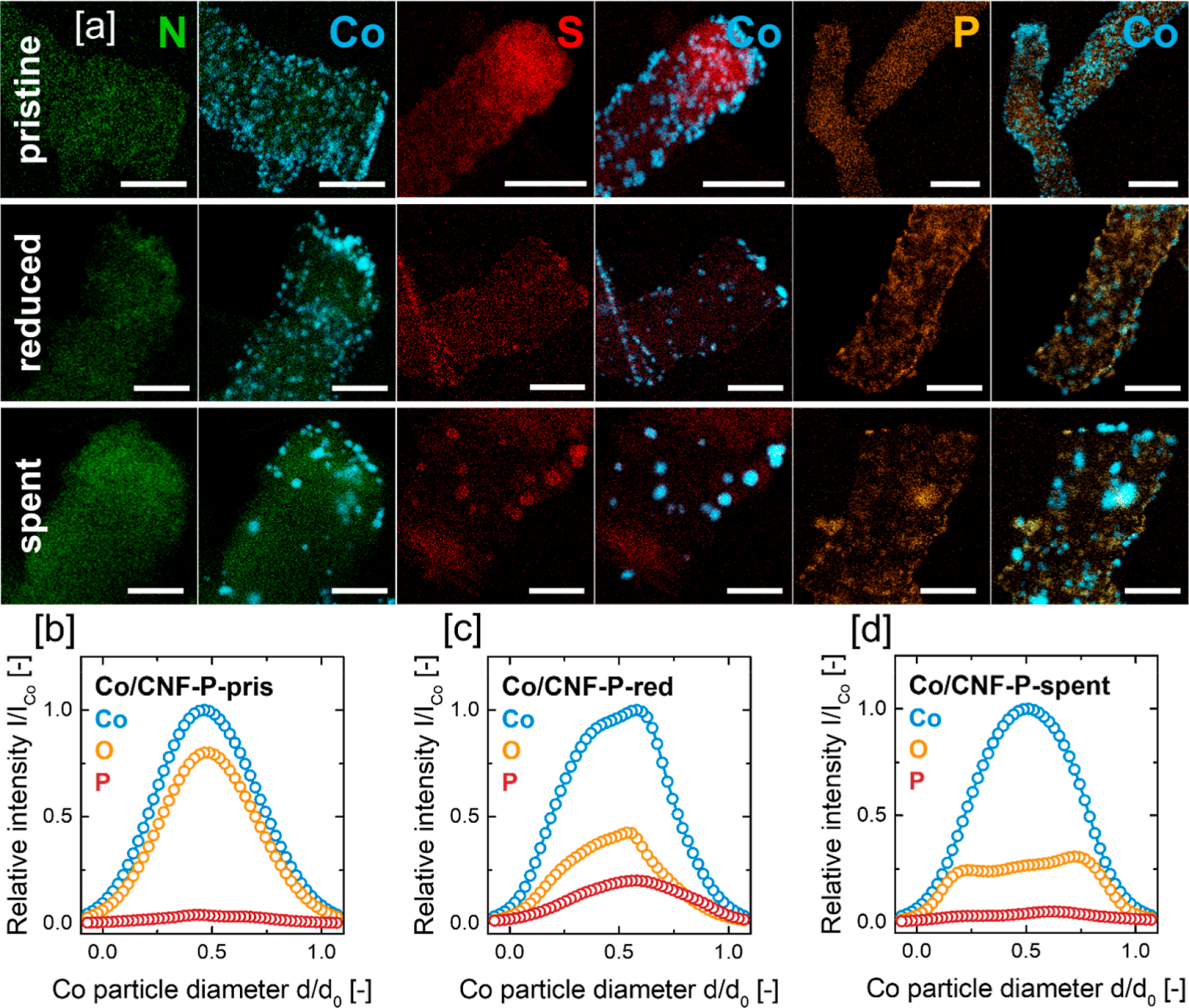
(a) EDS elemental maps of pristine, reduced, and passivated as well as spent (after 80 h FTS) and passivated Co/CNF catalysts. The scale bar corresponds to 100 nm. Averaged EDS line scans of 5–10 Co nanoparticles of (b) pristine Co/CNF-P, (c) reduced and passivated Co/CNF-P, and (d) spent and passivated Co/CNF-P.

XPS survey spectra of the doped and nondoped CNF supports indicated that the support surfaces were solely composed of C, O, and the respective heteroatom species (Figure S4). The oxygen content was found to be 2.3 at. % for CNF-H, 1.1 at. % for CNF-N, 1.8 at. % for CNF-S, and 4.0 at. % for CNF-P, suggesting that heteroatom doping with trimethyl phosphite (P(OCH_3_)_3_) as the phosphorus source yielded at least partially oxidized P species despite being carried out in a H_2_ atmosphere at elevated temperature (Table S1). Further qualitative and quantitative analysis was carried out by analysis of the XPS N 1s, S 2p, and P 2p regions of the respective heteroatom-doped carbon supports (Figure 1d–f). In case of CNF-N, pyridinic, pyrrolic, quaternary, and oxidized N species were identified, with pyridinic N being the most abundant (54%) followed by quaternary (20%) and pyrrolic N (18%). The S profile of CNF-S was found to be dominated by thiophenic S species (89%), with a minor contribution (10%) of oxidized species such as sulfones and sulfoxides. Lastly, analysis of the XPS P 2p contribution of CNF-P indeed revealed the presence of partially oxidized P species, whereas two P species of different oxidation states were identified. The binding energy of the reduced P species corresponded well to a stoichiometry of C_3_PO (using triphenylphosphine oxide as a reference),^48^ while the P species of a higher state of oxidation showed binding energies matching stoichiometries such as C_0–1_PO_3–4_,^49,50^ suggesting P species that either exhibit only one direct bond to C or are connected to the carbon surface by a bridging oxygen. The reduced P species made up 68% of the overall amount of P, while P in the higher state of oxidation accounted for 32%. The spatial distribution of N, S, and P was examined by STEM–EDS mapping, whereas the dopants were homogeneously distributed in all cases (Figure 2a; see Figures S5–S7 for enlarged EDS maps). In summary, employing gasification-assisted heteroatom doping allowed the controlled doping of CNF with N, S, and P while retaining the texture and nanostructure of the parental CNF (for further data and discussion of the GAHD approach, see ref (30)). The resulting series of doped and nondoped model catalyst supports thus differed predominantly in the presence or absence of heteroatom dopants, providing the basis to examine the isolated effects of carbon heteroatom doping on catalyst performance.

### 3.2 Pristine Co/CNF Catalysts

**Figure 3:**
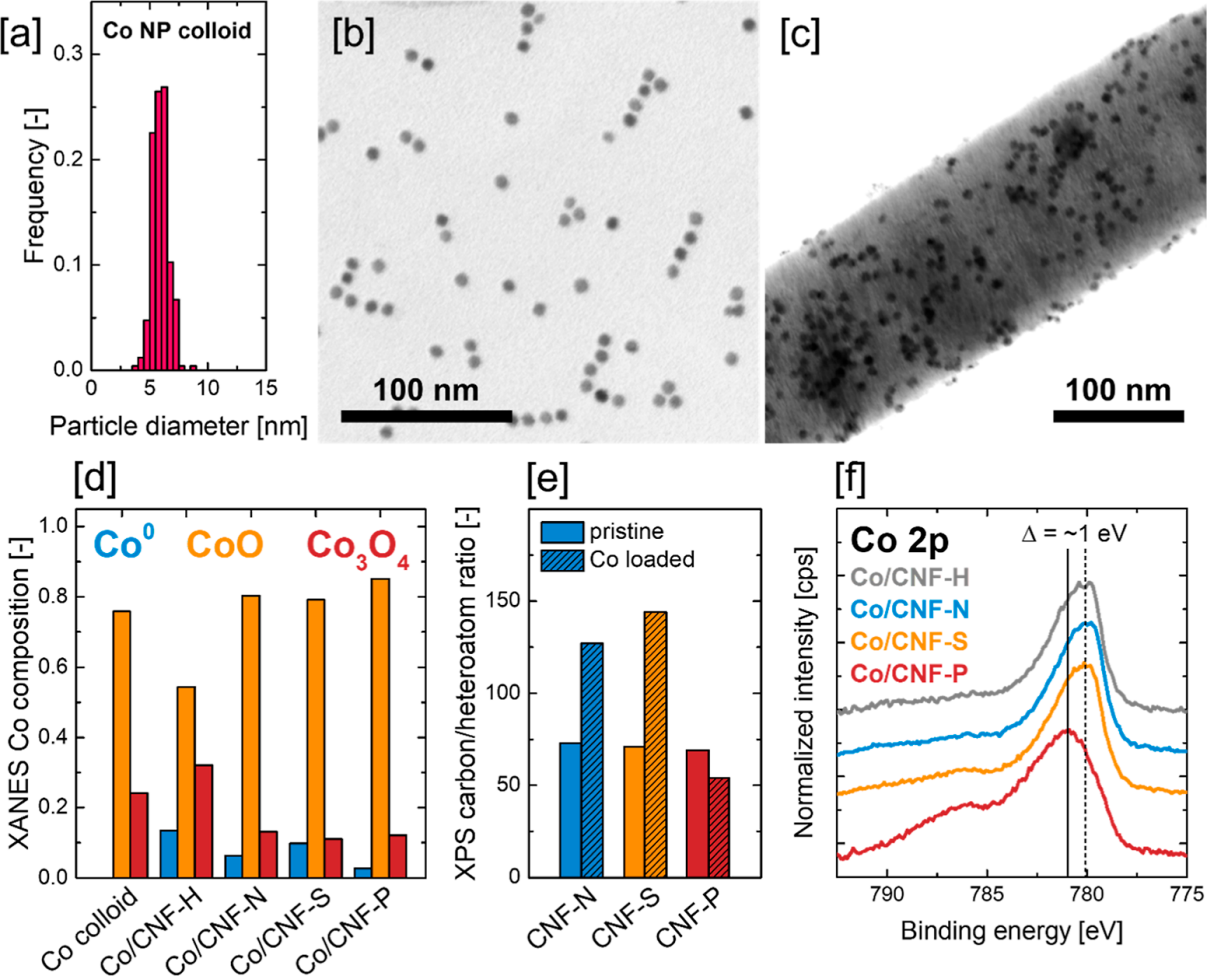
(a) Particle size distribution of colloidal cobalt oxide nanoparticles. STEM brightfield images of colloidal Co nanoparticles (b) before and (c) after loading on the different carbon nanofiber supports. (d) Composition of the Co nanoparticles before and after deposition on the CNF supports as determined by XANES-LCF analysis. (e) XPS carbon-to-heteroatom ratio of CNF-N, CNF-S, and CNF-P before and after Co loading. (f) XPS Co 2p_3/2_ contribution of the pristine Co/CNF catalysts.

Colloidal Co nanoparticles were prepared by thermal decomposition of Co_2_(CO)_8_ in the presence of oleic acid as a ligand. After exposure to air at room temperature, STEM imaging indicated a narrow size distribution for the cobalt oxide colloid, yielding an average cobalt oxide particle diameter of 7.4 ± 0.7 nm (Figure 3a,b). For the sake of comparability, Co nanoparticle diameters will be reported for metallic Co^0^ in the following, corrected for a 3 nm cobalt oxide surface layer (see Section 2.6 for details). Aiming for cobalt loadings around 6 wt %, cobalt nanoparticle attachment to the different CNF supports was carried out by wet impregnation, which afforded a homogeneous Co NP deposition without any significant aggregation (Table 1, Figures 3c and S8). The impregnation procedure did not change the Co particle size distribution, as STEM imaging found identical size distributions before and after loading of the different CNF supports (Figures S8 and S9). XANES LCF analysis showed that the Co nanoparticles in the colloid were composed of a mixture of CoO (76 at. %) and Co_3_O_4_ (24 at. %), while wet impregnation of the CNF supports at elevated temperatures (200 °C) and in N_2_ atmosphere appeared to cause partial reduction of the Co nanoparticles (Table 1, Figures 3d and S10). In this context, minor amounts of Co^0^ are detected for all Co/CNF samples, although CoO remains the dominant phase in all cases. XRD analysis of the pristine Co/CNF catalysts confirms this finding, detecting CoO as the dominant phase of the supported Co nanoparticles (Figure S11). The CoO crystallite size obtained by Rietveld refinement (3.2–3.9 nm) falls significantly short of the average Co particle diameter detected by STEM imaging (7.4 ± 0.7 nm, in the oxidized state), indicating polycrystalline Co nanoparticles, which compares well with previous literature reports (Table 1).^34^

**Table 1:** Properties of the Pristine Co/CNF Catalysts

sample	Co loading^a^ [wt %]	*D* (Co^0^)^b^ [nm]	Co^0^ fraction^c^ [at. %]	CoO fraction^c^ [at. %]	Co_3_O_4_ fraction^c^ [at. %]	CoO crystallite^d^ size [nm]
Co NP colloid		5.9 ± 0.7	0	76	24	
Co/CNF-H	5.6	5.9 ± 0.7	13	54	32	3.2
Co/CNF-N	6.3	5.9 ± 0.7	7	80	13	3.9
Co/CNF-S	5.6	5.8 ± 0.8	10	79	11	3.6
Co/CNF-P	5.0	5.8 ± 0.7	3	85	12	3.7

a As determined by MP-AES.

b Average Co^0^ particle diameter. Obtained by STEM imaging, corrected for a 3 nm oxide layer.

c Determined by XANES LCF analysis.

d Determined by Rietveld analysis of XRD patterns.

By comparing the ratio of carbon surface atoms to N, S, or P surface atoms (C/X ratio, determined by XPS) before and after Co loading, preferential adsorption of the Co NPs on the heteroatom-doped carbon nanofibers was studied (Figure 3e). In case of preferential adsorption on the heteroatom species, the C/X ratio is expected to increase as the covering of the heteroatom surface species by Co nanoparticles prevents detection by surface-sensitive XPS. It should be noted that the preferential adsorption of Co NPs on carbon surface oxides is also possible. However, the oxygen species introduced by the loading of cobalt oxide NPs overlap with the carbon surface oxides in the XPS O 1s spectrum, impeding any reliable determination of the C/O ratio, where oxygen originates exclusively from the carbon surface species, after the loading of Co NPs. In case of CNF-N and CNF-S, a clear increase of the C/X ratio could be detected upon Co loading (from 73 to 127 for CNF-N, from 71 to 144 for CNF-S), indicating preferential adsorption of Co nanoparticles on N and S surface species. For CNF-P, the C/X ratio decreased by a small margin after cobalt loading (from 69 to 54), suggesting a random adsorption of Co nanoparticles on the surface of CNF-P. In all cases, the relative amount of individual N, S, and P species (e.g., the ratio of pyridinic to pyrrolic to quaternary N) remained comparable before and after Co loading (Figure S12).

Analysis of the XPS Co 2p region of the pristine Co/CNF catalysts showed similar 2p_3/2_ bands and satellites for Co/CNF-H, Co/CNF-N, and Co/CNF-S featuring 2p_3/2_ binding energies around 780.0 eV (Figure 3f). For Co/CNF-P, the 2p_3/2_ band of Co was found to be shifted to higher binding energies around 781.0 eV and additionally showed a pronounced satellite centered around 786 eV. As the binding energies of CoO (2p_3/2_ 780.7 eV) and Co_3_O_4_ (2p_3/2_ 780.1 eV) are located in this range, an explanation for the shift in the p_3/2_ maximum might be found in a difference in the cobalt surface composition, with Co NPs on CNF-P exhibiting a CoO-rich surface, while the cobalt surfaces on the other supports are rich in Co_3_O_4_.^51^ This interpretation is corroborated by the distinct satellite band in the Co 2p_3/2_ spectrum of Co/CNF-P at 786 eV, which is associated with Co(II) species, while Co(III) species are known to exhibit less-pronounced satellites.^52^ However, it should be noted that XANES-LCF analysis yielded comparable Co phase compositions at least for Co/CNF-N, Co/CNF-S, and Co/CNF-P, suggesting differences in surface and bulk composition of the Co nanoparticles. A second reason for the shift of the Co 2p_3/2_ maximum for Co/CNF-P might be charge transfer from Co to the support, caused by the high surface concentration of electronegative species (O, P) on the catalyst support. However, given the average CoO nanoparticle diameter of 7.4 ± 0.7 nm and the spherical particle morphology observed by STEM imaging, the ratio of “bulk” cobalt atoms to Co/support interface atoms is expected to be high, attenuating the effects of charge transfer significantly. Nevertheless, there are literature studies that were able to probe charge transfer effects between larger Pd nanoparticles (∼5 nm) supported on carbon nanotubes,^53^ rendering both charge transfer and differences in the Co NP surface composition possible explanations for the shift in binding energy of the Co 2p_3/2_ contribution of Co/CNF-P.

### 3.3 Catalyst Reduction

**Figure 4:**
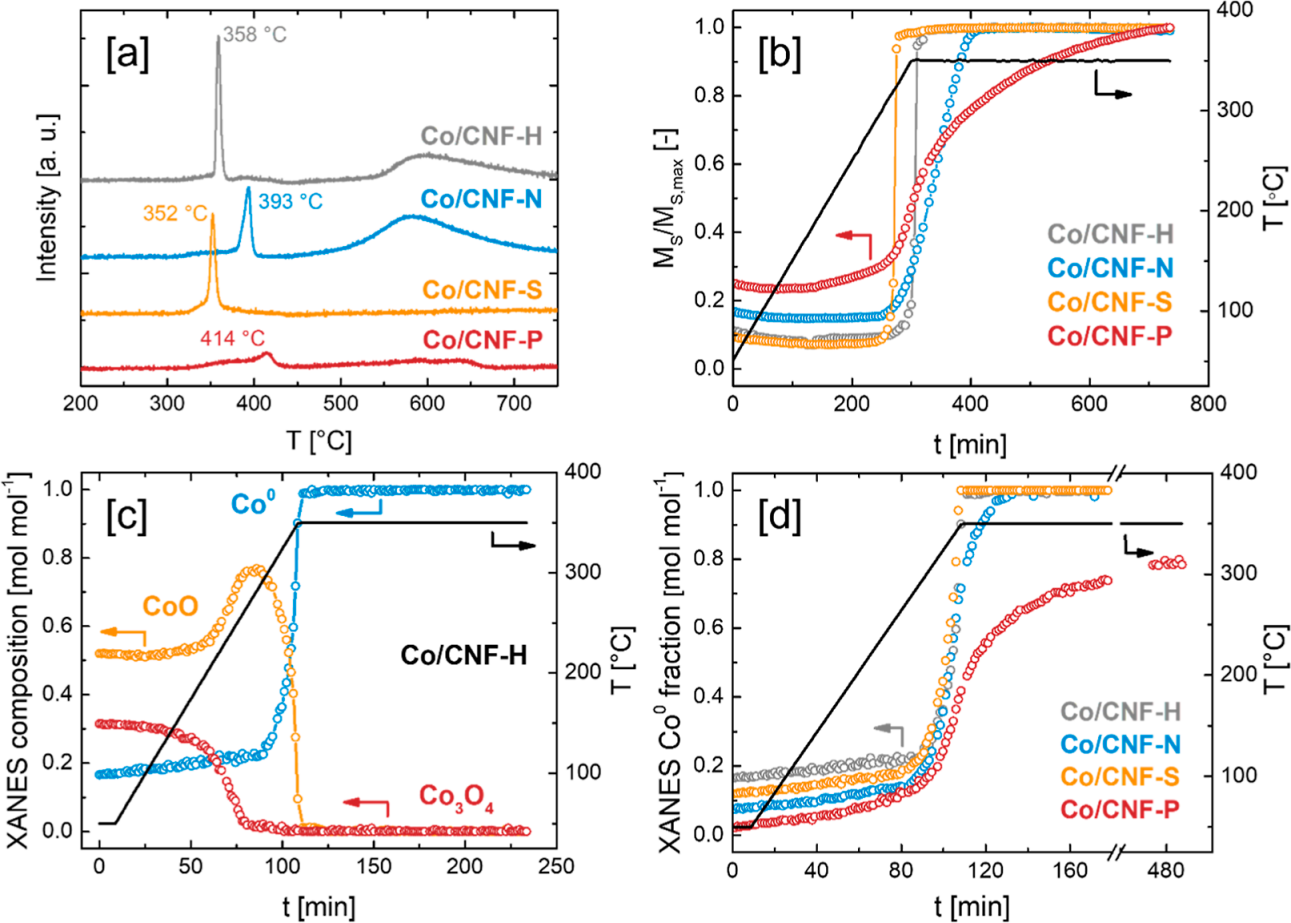
(a) H_2_-TPR of Co/CNF catalysts. (b) In situ measurement of the saturation magnetization at 2.0 T of the Co/CNF catalysts during catalyst reduction (1 °C min^–1^ to 350 °C, 25 vol % H_2_ in Ar). *M*_S_ is normalized to the saturation magnetization at the end of the experiment. (c) In situ Co K-edge XANES-LCF analysis of the Co phase composition of Co/CNF-H during reduction (3 °C min^–1^ to 350 °C, 25 vol % H_2_ in He). (d) Comparison of the Co^0^ fraction of all Co/CNF catalysts during reduction (3 °C min^–1^ to 350 °C, 25 vol % H_2_ in He), derived from in situ Co K-edge XANES-LCF analysis.

H_2_-TPR characterization of the Co/CNF catalysts revealed distinct differences in Co reducibility depending on the employed CNF support (Figure 4a). Co supported on the reference CNF-H showed two H_2_ uptake signals, a sharp peak at 358 °C that could be assigned to the one-step reduction of CoO to Co^0^ and a broad feature centered around 600 °C that was assigned to Co^0^-catalyzed methanation of the carbon support by van Deelen et al.^34^ Co/CNF-N showed a similar reduction profile, although Co reduction proceeded at higher temperature (393 °C), indicating stronger Co/support interactions as a consequence of N-doping. This compares well to some literature reports;^16,17,21^ however, some studies^13,20^ also reported an increased Co reducibility on N-doped carbon as compared to a nondoped support. The reason for those contradictory reports may be found in poorly comparable Co particle size distributions on doped and nondoped carbon supports as Co reducibility is known to be particle size dependent.^54^ Co/CNF-S exhibited a slightly lower Co reduction temperature (352 °C) compared to the reference Co/CNF-H and showed no sign of carbon methanation at elevated temperatures, hinting at a possible interference of the S species in this process. In contrast to the other Co/CNF catalysts, an undefined, broad Co reduction peak centered at 414 °C was observed for Co/CNF-P, indicating strong metal/support interactions and in consequence a low Co reducibility supported on CNF-P. At higher temperatures, some carbon support methanation was detected over Co/CNF-P, although at a lower rate compared to Co/CNF-H and Co/CNF-N.

In order to gain further insights into Co reducibility on the different supports, in situ magnetometry and in situ XAS experiments were conducted. For the in situ magnetometry experiments, the saturation magnetization in an external magnetic field of 2 T was measured, which correlates directly to the amount of Co metal in the reactor as only Co^0^ displays an appreciable magnetic susceptibility and thus contributes to saturation magnetization (CoO and Co_3_O_4_ display very low magnetic susceptibilities; see Experimental section for further details) (Figure 4b). For the in situ magnetometry experiments, Co/CNF reduction was conducted under identical experimental conditions as used for catalyst reduction before FTS catalytic testing (1 °C min^–1^ to 350 °C, 8 h hold at 350 °C, 25 vol % H_2_ in Ar). In situ magnetometry confirmed the general trends observed in H_2_-TPR, with Co reducibility decreasing in the order of Co/CNF-S > Co/CNF-H > Co/CNF-N > Co/CNF-P. While Co reduction over the CNF-S and CNF-H supports proceeded rapidly once the reactor temperature exceeded 330 °C, saturation magnetization for Co/CNF-N and Co/CNF-P increased significantly slower. While Co/CNF-N reached stability after ∼90 min at 350 °C, the saturation magnetization of Co/CNF-P was still slowly increasing even after 8 h of reduction at 350 °C.

In addition to in situ magnetometry, the reducibility of the Co/CNF catalysts was probed by in situ XANES at the Co K-edge (Figures 4c,d and S13). For an efficient use of beam time, the experimental conditions of the reduction experiments were adapted (3 °C min^–1^ to 350 °C, 1.5–6 h hold at 350 °C, 25 vol % H_2_ in He). XANES LCF analysis yielded the typical two-step Co reduction mechanism starting with the conversion of Co_3_O_4_ to CoO in the temperature range between 160 and 260 °C, followed by the reduction of CoO to Co^0^ starting around 290 °C for all Co/CNF catalysts. While Co/CNF-H, Co/CNF-N, and Co/CNF-S attained a degree of reduction (DOR) of 1 shortly after the temperature reached 350 °C, Co supported on CNF-P showed a lower reducibility, leveling out at a DOR of 0.79 after 6 h at 350 °C. Regarding the impeded reducibility of Co/CNF-P, we hypothesize that strong interactions between surface phosphorus species on CNF-P and cobalt oxide nanoparticles restrict reduction to areas of the Co nanoparticles that are not in close proximity to the support surface.

### 3.4 Reduced Catalysts

**Figure 5:**
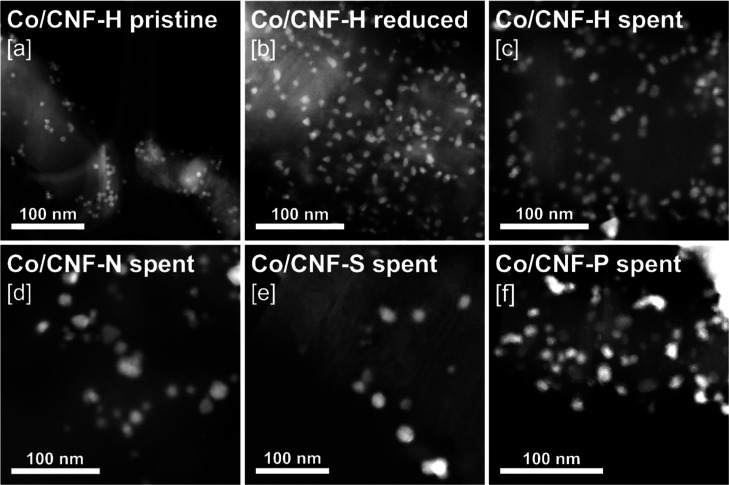
STEM HAADF images of (a) pristine Co/CNF-H, (b) reduced and passivated Co/CNF-H, (c) spent (after 80 h FTS) and passivated Co/CNF-H, (d) spent and passivated Co/CNF-N, (e) spent and passivated Co/CNF-S, and (f) spent and passivated Co/CNF-P.

**Figure 6:**
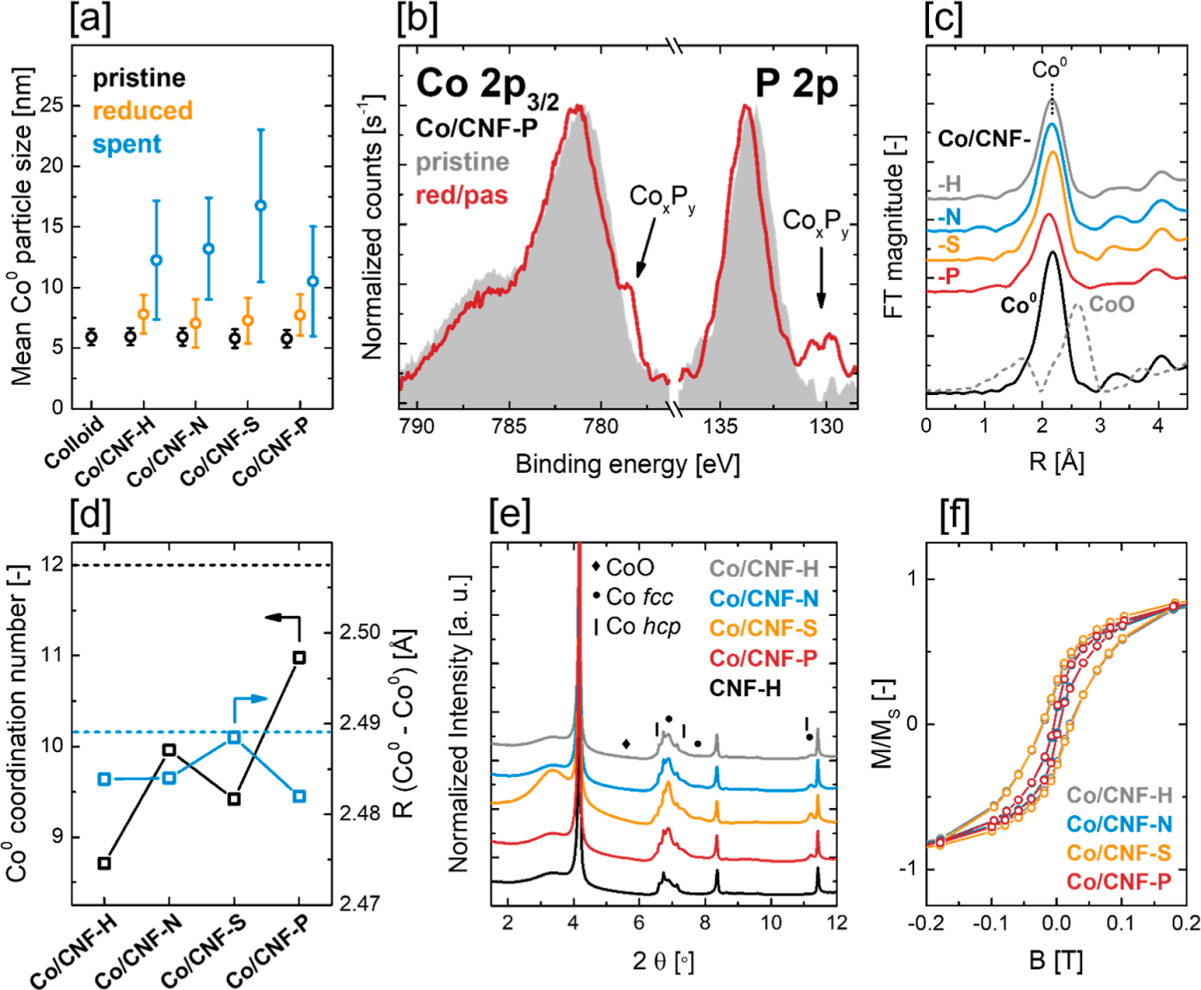
(a) Average Co^0^ nanoparticle size of the pristine, reduced, and spent Co/CNF catalysts. (b) Comparison of the XPS P 2p and Co 2p_3/2_ regions before and after reduction/passivation of Co/CNF-P. (c) *k*^2^-weighted in situ EXAFS data of the reduced Co/CNF catalysts in the *R* space. (d) First shell Co^0^ coordination numbers and Co^0^–Co^0^ interatomic distances of the reduced catalysts. (e) In situ XRD patterns of the reduced Co/CNF catalysts. (f) Magnetic hysteresis measured in situ at 350 °C in 25 vol % H_2_ in He of the reduced Co/CNF catalysts.

The reduced catalysts were studied by STEM imaging, STEM–EDS, XPS, magnetometry, XRD, and EXAFS. It should be noted that STEM imaging, STEM–EDS, and XPS were conducted after catalyst reduction, passivation, and air exposure, while magnetometry, XRD, and EXAFS were executed in situ without air exposure after the respective reduction runs. STEM imaging of the reduced and passivated Co/CNF catalysts showed that the average Co nanoparticle size increased uniformly for all catalysts from 5.8–5.9 nm to 7.0–7.8 nm, suggesting that Co NP growth during reduction was not significantly influenced by the presence or absence of the heteroatom dopants (Figures 5, 6a, S14, and S9). STEM–EDS mapping indicated that the N dopant remained homogeneously distributed after reduction and passivation; however, in case of Co/CNF-S and Co/CNF-P, some spatial redistribution of S and P species could be observed. In this context, catalyst reduction appeared to promote the concentration of S and P around the Co nanoparticles. Averaged EDS line scans of 5–10 individual Co NPs clearly showed that the P concentration of the Co NPs increased significantly upon reduction of Co/CNF-P (Figure 2b,c). This suggests that either Co nanoparticles migrate across the support surface and “collect” phosphorus species during their movement or that the phosphorus species themselves exhibit mobility. The interaction between Co and the support dopant could be corroborated by XPS, as cobalt phosphide surface species could be detected in both Co and P contributions (Co 2p_3/2_ binding energy 778.7 eV, P 2p_3/2_ binding energy 129.8 eV) after catalyst reduction and passivation (Figure 6b).^55^ Deconvolution of the P 2p contribution revealed a loss of the reduced C_2–3_PO_1–2_ species relative to the oxidized C_0–1_PO_3–4_ species, indicating that the reduced P species was converted to Co_*x*_P_*y*_ during catalyst reduction (Figure S15). In addition to nonreducible cobalt oxides, the cobalt phosphide phase is likely contributing to the low Co reducibility observed for Co/CNF-P. For reduced and passivated Co/CNF-S and Co/CNF-N, no new cobalt/dopant compounds could be detected by XPS, while the Co 2p_3/2_ binding energy maxima (between 780.7 eV for Co/CNF-N to 781.1 eV for Co/CNF-P) as well as the distinct satellites centered around 786 eV indicated CoO as the dominant Co surface species for all reduced and passivated catalyst samples (Figure S16).^51,52^

In situ EXAFS characterization was carried out by fitting of the first coordination shell of the reduced catalysts (Figures 6c,d and S17–S19, Table S2). In all cases, Co^0^–Co^0^ scattering path lengths did not deviate by more than 0.01 Å from a Co hcp foil standard, which is within the expected accuracy of the measurement of ±0.02 Å. However, the first shell coordination numbers (CN) of some reduced Co/CNF catalysts showed significant deviation from the Co standard (CN = 12 for bulk Co, uncertainty of the measurement typically ±10%), with only Co/CNF-P (CN 11.0) showing a coordination number identical with that of the standard within the range of error. Coordination numbers of Co/CNF-H (CN 8.7), Co/CNF-N (CN 10.0), and Co/CNF-S (CN 9.4) were found to be significantly smaller than expected. In this context, the average Co^0^ nanoparticle diameters around ∼7.5 nm are well above the nanoparticle size threshold (∼5 nm), below which an increasing fraction of surface atoms results in a decrease in coordination number.^56,57^ This mismatch between Co nanoparticle size and coordination number was observed before for supported Co catalysts,^57−60^ whereas surface disorder^61^ and/or structural disorder such as polycrystallinity^59^ were discussed as possible explanations.

In situ XRD of the reduced Co/CNF catalysts was conducted to gain insight on Co phase composition and crystallite size (Figures 6e and S20, Table S3). All catalysts showed the presence of crystalline Co^0^ in form of an intergrowth structure of the Co hcp and Co fcc polymorphs. Other crystalline phases could not be detected in any sample, although in situ XANES and EXAFS suggested the presence of 21 wt % of CoO in the reduced Co/CNF-P sample, while XPS indicated the presence of Co_*x*_P_*y*_ species. It should be noted that EXAFS analysis was attempted with a Co_2_P standard but did not yield satisfying fits. The diffraction patterns of the Co^0^ phase matched well with a so-called hcp/fcc intergrowth phase in all samples.^62^ This means that Co NPs consist of intergrown fcc and hcp domains, which can be distinguished from a mixture of phase-pure fcc and hcp crystallites by characteristic relative peak intensities in XRD.^62^ In this context, Co nanoparticles may exhibit polycrystallinity as crystallite sizes derived by XRD are significantly smaller than the Co particle size derived by microscopy techniques.^63^ This Co intergrowth phase is well-known to form during reduction of supported Co nanoparticles in hydrogen and was encountered in wide variety of studies.^34,56,62,64−66^ Due to the presence of stacking disorder associated with intergrown fcc/hcp domains, Rietveld refinement of this phase assuming a simple mixture of the pure fcc and hcp polymorphs yielded inadequate results. In this context, a Rietveld refinement procedure of van Deelen et al. was adopted, which achieves better refinement results by assuming the preferred orientation of Co hcp crystallites.^34^ This assumption may be physically disputable and contributes to a higher uncertainty of the refinement results; however, it enables an adequate reproduction of the diffraction features. This refinement procedure afforded similar hcp/fcc ratios of roughly 70:30 for Co/CNF-H, Co/CNF-S, and Co/CNF-P; only for Co/CNF-N, it was found to be 30:70 (Figure S20, Table S3). It should be noted that support properties and Co^0^ phase composition are likely interconnected. As Co reduction takes place in the presence of the doped/nondoped carbon supports, differences in phase composition after reduction likely arise as a consequence of variations in the support properties. While Co fcc crystallite sizes were found to be in the range of the Co nanoparticle sizes determined by STEM (∼7.5 nm) for all samples, crystallite sizes of the hcp polymorph were significantly smaller, which was previously assigned to be an artifact of the assumption of preferred crystal orientation for Co hcp.^34^ However, Sławiński et al. proposed to use the (overlapping) Co^0^ (110)hcp/(022)fcc diffraction peak around 11.2°/2θ for an fwhm-based evaluation of Co crystallite size (e.g. following the Scherrer equation) as it is not affected by the stacking disorder induced by fcc/hcp intergrowth.^62^ Determination of the fwhm of the (110)hcp/(022)fcc diffraction peak yielded very similar values of 0.15–0.16°/2θ for all catalysts, suggesting that the Co crystallite size was comparable for all samples (Figure S20e).

In situ characterization of the reduced Co/CNF catalysts by magnetometry showed differences in the magnetic hysteresis behavior, with Co/CNF-H and Co/CNF-S exhibiting a higher remanent magnetization as compared to Co/CNF-N and Co/CNF-P (Figures 6e and S21; see Section 2.7 for details). Considering the very similar Co nanoparticle size after catalyst reduction, those differences may be induced by polycrystallinity of Co/CNF-N and Co/CNF-P, with Co nanoparticles on CNF-N and CNF-P consisting of multiple smaller crystal/magnetic domains with dimensions below the critical diameter *D*_c_, which consequently exhibit superparamagnetism. However, in situ XRD does not support this explanation as all catalysts were found to exhibit similar Co crystallite size (Figure S20e, Table S3). On the other hand, it is known that the critical diameter above which remanent magnetization occurs depends on the crystal structure, as Bean and Livingston determined D_c_ for Co hcp to be 8 nm and for Co fcc to be 28 nm.^46^ In situ XRD suggested Co/CNF-N to be Co fcc rich (28:72 hcp/fcc) as opposed to Co/CNF-H (68:32 hcp/fcc) and Co/CNF-S (69:31 hcp/fcc) being Co hcp rich (Figure S20f, Table S3), providing a plausible explanation for a higher fraction of superparamagnetic Co in case of Co/CNF-N. The situation is more complex for Co/CNF-P, as Rietveld refinement of in situ XRD indicated a hcp-rich Co phase (73:27 hcp/fcc); however, the magnetic behavior may be influenced by the presence of nonmetallic Co species, namely, the noncrystalline CoO phase suggested by in situ XANES and in situ XRD, as well as the Co_*x*_P_*y*_ phase observed by XPS.

### 3.5 Fischer–Tropsch Synthesis

**Figure 7:**
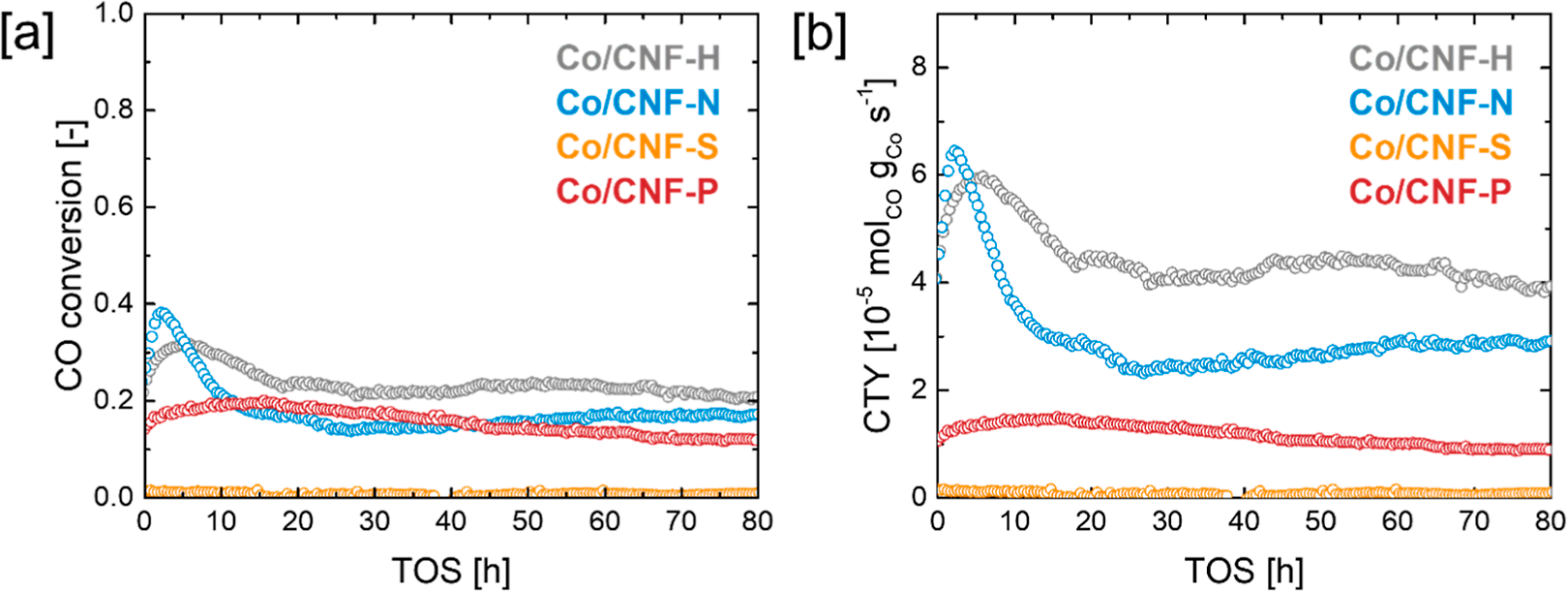
Fischer–Tropsch synthesis activity in terms of (a) CO conversion and (b) CTY over 80 h time on stream (220 °C, 20 bar, H_2_/CO 2.1, WHSV 1.2–3 L g_Cat_^–1^ h^–1^).

After catalyst reduction at 350 °C for 8 h, FTS was conducted for 80 h time on stream at 220 °C, 20 bar and a syngas ratio (H_2_/CO) of 2.1 (Figure 7, Table 2). To achieve similar CO conversion in the range of 20–30%, WHSVs were varied between 1.2 L g^–1^_Cat_ h^–1^ and 3 L g^–1^_Cat_ h^–1^ depending on catalyst activity. Catalyst activity is reported as CO consumption normalized to the Co mass (CTY, cobalt time yield), with Co/CNF catalysts displaying significantly different activities depending on the presence or absence of heteroatom dopants (see Figure S22 for site time yields). Co/CNF-H and Co/CNF-N showed pronounced induction periods, during which an initial spike of activity peaking around 6.0–6.4 × 10^–5^ mol_CO_ g_Co_^–1^ s^–1^ was followed by a decline in CO conversion until pseudo steady-state was reached at activities of 4.5 × 10^–5^ mol_CO_ g_Co_^–1^ s^–1^ for Co/CNF-H and 2.9 × 10^–5^ mol_CO_ g_Co_^–1^ s^–1^ for Co/CNF-N around 17 h TOS. Co/CNF-P did not show a pronounced initiation period with a CTY of 1.5 × 10^–5^ mol_CO_ g_Co_^–1^ s^–1^ at 17 h TOS. Co/CNF-S showed only negligible FTS activity. Comparison of the C_5+_ selectivities at similar conversion level (17 h TOS, 17–24% CO conversion) showed a similar trend as FTS activity, with Co/CNF-H displaying the highest C_5+_ selectivity of 66%, followed by Co/CNF-N (64%) and Co/CNF-P (54%, see Table S4 and Figure S23 for further selectivity data). CO_2_ selectivity was found to be negligible, for example, ≤1% for all catalyst samples. Likewise, no evidence for the formation of significant amounts of oxygenates was found. In all cases, catalyst deactivation was significant, with Co/CNF-H losing 34% of its maximum CTY over 80 h TOS, followed by Co/CNF-P with −38% and the most severe loss of activity being observed for Co/CNF-N with −58%.

**Table 2:** FTS Activity and Selectivity of Co/CNF Catalysts^a^

sample	*X*_CO,m_ [%]	CTY_m_^b^	*X*_CO,ai_ [%]	CTY_ai_^b^	*S*_CH_4_,ai_ [%_C_]	*S*_C_*5*+_,ai_ [%_C_]	CTY_f_^b^	*S*_CH4,f_ [%_C_]	*S*_C_5+_,f_ [%_C_]
Co/CNF-H	32	6.0	24	4.5	21	66	3.9	27	58
Co/CNF-N	38	6.4	17	2.9	27	60	2.9	24	64
Co/CNF-S	1	0.1	1	0.1			0.1		
Co/CNF-P	20	1.5	20	1.5	29	54	0.9	35	46

a _m_ maximal conversion/CTY reported between 0 and 15 h TOS. _ai_ after induction, reported at comparable conversion after the initiation period at ∼17 h TOS. _f_ final, reported at 80 h TOS.

b Cobalt time yield, reported as 10^–5^ mol_CO_ g_Co_ s^–1^.

Considering the difference in FTS activity relative to the reference Co/CNF-H, both Co/CNF-S and Co/CNF-P seem to experience catalyst poisoning. In this context, Co/CNF-S exhibited negligible activity due to sulfur poisoning, while Co/CNF-P showed significantly reduced FTS activity, likely due to the formation of the Co_*x*_P_*y*_ phase and comparatively poor cobalt reducibility (DOR 0.79). Variations in catalytic performance may also be related to differences in the Co hcp/fcc ratios (68:32 hcp/fcc for Co/CNF-H, 28:72 hcp/fcc for Co/CNF-N, Figure S20), with the hcp polymorph being more active for FTS.^66−68^ In this context, the higher initial activity of Co/CNF-N as compared to that of Co/CNF-H seems to contradict the general finding that the hcp polymorph displays higher FTS activity. However, it should be noted that in both cases, a complex Co hcp/fcc intergrowth structure is present, which is difficult to compare to phase-pure Co hcp or fcc model systems in terms of catalytic activity. As mentioned previously, the disordered nature of this intergrown structure additionally introduces significant uncertainty regarding the hcp/fcc ratios derived from Rietveld refinement of XRD patterns, meaning that the derived hcp/fcc ratios should be interpreted with caution. In any case, FTS activity is the consequence of multiple factors of influence in the complex systems investigated, of which the Co phase composition is only one. In this context, the high initial activity of Co/CNF-N might be the consequence of other factors, such as charge transfer between metal and support. Although charge transfer is generally considered a short-range metal/support interaction and the Co^0^ nanoparticles on Co/CNF-N and Co/CNF-H display large diameters in this context (∼7.5 nm after reduction), it is conceivable that Co surface atoms in close proximity to the Co/C interface experience electronic interactions with the N-doped support. This hypothesis is supported by the work of Cheng et al., who employed in situ XPS on reduced Co nanoparticles supported on various nitrogen-doped carbon nanospheres.^16^ Their findings suggested that significant charge transfer occurs on carbon materials with a high nitrogen content (up to ∼7 at. % N), resulting in an increased electron density of the Co phase. This electron-rich Co^0^ phase was associated with enhanced FTS activity. Additionally, the structural order of the Co nanoparticles may influence FTS activity. Co/CNF-H exhibited a lower first-shell Co coordination number, suggesting a higher degree of structural disorder (such as polycrystallinity),^59^ which is associated with lower FTS activity.^66^ However, due to the onset of catalyst deactivation for both Co/CNF-H and Co/CNF-N from the very start of the catalytic tests, meaningful activity comparisons remain challenging.

### 3.6 Catalyst Deactivation

**Figure 8:**
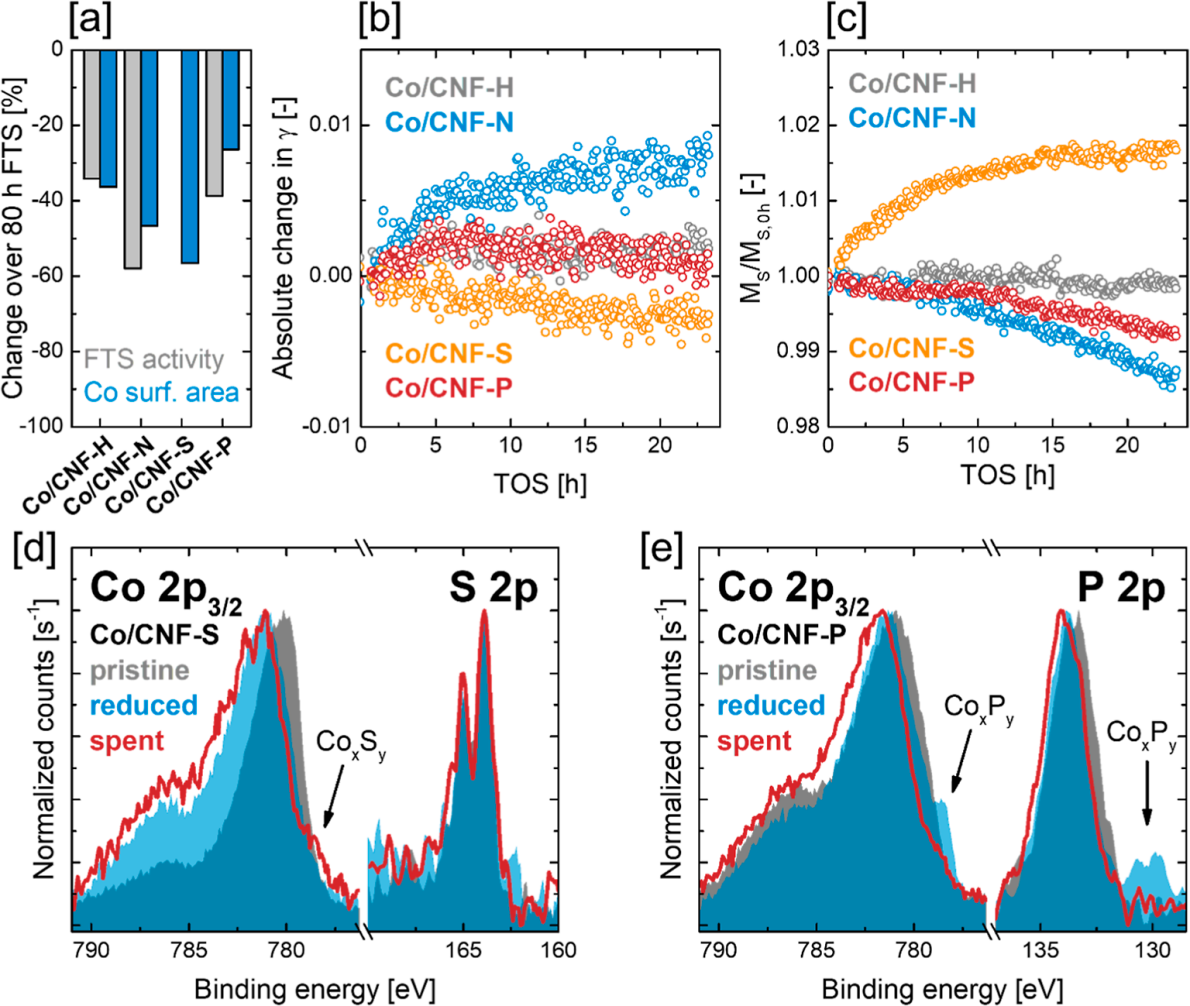
(a) Comparison of the loss in FTS activity with the loss in Co surface area after 80 h FTS. (b) In situ magnetometry monitoring of γ for all Co/CNF catalysts over 24 h FTS (220 °C, 20 bar, H_2_/CO 2.1). (c) In situ magnetometry monitoring of the saturation magnetization of all Co/CNF catalysts over 24 h FTS (220 °C, 20 bar, H_2_/CO 2.1). *M*_S_ is normalized to the saturation magnetization at the start of the experiment (*t* = 0 h). (d) Comparison of the XPS S 2p and Co 2p_3/2_ regions of pristine, reduced/passivated, and spent/passivated Co/CNF-S. (e) Comparison of the XPS P 2p and Co 2p_3/2_ regions of pristine, reduced/passivated, and spent/passivated Co/CNF-P.

Comparison of the loss of FTS activity and loss of Co surface area clearly showed that Co nanoparticle sintering was the dominant deactivation pathway in all catalysts (Figures 5, 6a and 8a). Compared to the reference Co/CNF-H (−36% Co surface area over 80 h TOS), only the presence of P appeared to inhibit Co particle growth (−26%), while the presence of N (−47%) and S (−57%) contributed to accelerated Co NP sintering. In light of negligible CO conversion, it is noteworthy that Co on CNF-S showed the highest sintering tendency (Co NPs growing from 7.3 ± 1.9 nm after reduction to 16.7 ± 6.3 nm after FTS), even though there was little exposure to H_2_O, which is known to facilitate Co sintering (Figure 6a).^69^ It is furthermore noteworthy that spent Co/CNF-P was the only sample exhibiting a significant fraction of Co nanoparticles smaller than 5 nm (Figure S9). Given that the average Co^0^ nanoparticle size of Co/CNF-P postreduction was 7.7 ± 1.7 nm, this implies that a subset of Co nanoparticles shrank considerably during FTS. This observation might be a consequence of Ostwald ripening, involving the migration of Co atoms or clusters from a set of shrinking Co NPs to a set of growing nanoparticles. For this mechanism to occur, the support surface must be capable of stabilizing migrating Co atoms or clusters, which may be facilitated by P or PO_*x*_ surface species on CNF-P. In terms of catalyst deactivation, Co/CNF-H showed a decline in FTS activity that matched the loss of Co surface area very well, indicating that Co particle growth was the only relevant deactivation mechanism. For Co/CNF-N and Co/CNF-P, the loss of FTS activity significantly surpassed the loss of Co surface area, indicating that further deactivation pathways are at play (Figure 8a).

In situ monitoring of Co particle growth during FTS was attempted via magnetometry, using the γ parameter, which indicates the fraction of Co particles exceeding the critical diameter *D*_c_ (Figure 8b). γ is expected to detect Co particle growth, if sintering causes nanoparticles diameters to cross the threshold value of *D*_c_, elevating the actual value of *D*_c_ to a high importance for the sensitivity of gamma. While γ was monitored for all catalysts for the first 24 h TOS at 220 °C, 20 bar, and a syngas ratio of 2.1, it proved to be relatively insensitive to Co particle growth. Even though significant sintering was observed by STEM imaging for all catalysts, γ, for example, the fraction of Co particles larger than *D*_c_, increased by less than 1%. This observation is hard to interpret as it may suggest that the actual value of *D*_c_ for all catalysts significantly exceeded the size range of Co NPs, even though all catalysts showed at least some remanent magnetization after reduction, indicating that a fraction of Co NPs exhibited indeed diameters larger than *D*_c_ (see Co NP size distributions, Figure S9). Alternatively, this result might simply indicate that the magnetic domain sizes did not change significantly upon sintering of Co NPs. Qualitatively, the most significant change in γ was observed in the first 10 h TOS for Co/CNF-N, which coincided with a stage of rapid FTS activity loss (Figure 7), suggesting that Co NP sintering contributed to this stage of fast catalyst deactivation.

In situ measurements of the saturation magnetization *M*_S_ were used to indicate Co phase transformations as further deactivation pathway (Figure 8c). In this context, only the catalytically active Co^0^ phase exhibits a high magnetic susceptibility, meaning that the formation of other Co species leads to a decrease in saturation magnetization (see Section 2.7 for details). For the reference Co/CNF-H, the saturation magnetization remained stable over 24 h TOS at 220 °C, 20 bar, and a syngas ratio of 2.1. This fits well with the comparison of FTS activity loss and loss of Co surface area, which indicated that Co particle growth was the only relevant deactivation pathway and Co phase transformations did not play a role. On the other hand, for Co/CNF-N and Co/CNF-P, a steady decline in saturation magnetization over 24 h TOS clearly indicated that catalytically active Co^0^ was transformed to other, FTS-inactive Co species, contributing to catalyst deactivation. Even though the loss of Co^0^ was observed to be only around 1.5% over 24 h TOS for Co/CNF-N, Co phase transformation may nevertheless contribute significantly to catalyst deactivation considering that the fraction of surface atoms for spherical Co nanoparticles with a diameter of 13.2 nm [mean Co NP size of spent Co/CNF-N (Figure 6a)] is only around 7%. Despite clear signs of sulfur poisoning, the amount of Co^0^ appeared to increase over 24 h FTS for Co/CNF-S. Given that the degree of reduction was determined to be 100% by in situ XAS, a possible explanation for this observation may lie in the severe sintering observed for Co/CNF-S, which decreases the fraction of Co surface atoms available for chemisorption of S species. Given the positive influence of S species on Co reducibility (Figure 4) and the highly reducing conditions at negligible conversions, a reduction of Co_*x*_S_*y*_ surface species to Co^0^ bulk species during Co NP sintering seems conceivable.

STEM–EDS and XPS of the spent and passivated catalysts were conducted to further characterize Co phase transformations occurring during FTS. STEM–EDS mapping of spent Co/CNF-N showed that no redistribution of N species took place during FTS (Figures 2a and S5), suggesting that the Co phase transformation observed via in situ magnetometry may be assigned to the formation of cobalt oxides or to the formation of Co_*x*_N_*y*_ species that are not stable in air. A reason for the assumed deactivation of Co/CNF-N by the formation of cobalt oxides may be found in the differences in the electronic structure, as Cheng et al. found via in situ XPS that Co supported on N doped carbon exhibited significantly higher electron density as compared to Co NPs on a nondoped carbon support.^16^ Interestingly, Wolf et al. have demonstrated that oxidation and sintering of Co nanoparticles are interconnected under FTS conditions on carbon catalyst supports.^69^ In this context, oxidized Co nanoparticles appear to exhibit increased mobility, which enhances their sintering rate via particle migration and coalescence pathways as compared to fully reduced Co nanoparticles. This interconnectivity may also play a role in the deactivation of Co/CNF-N, as Co NP sintering and Co phase transformation are observed concurrently. In case of spent Co/CNF-S, a distinct spatial redistribution of the S species is observed in STEM–EDS elemental maps (Figures 2a and S6), with high concentrations of S coinciding with the location of Co nanoparticles, thus indicating substantial S poisoning of the catalyst. It should be noted that the reason for S poisoning is most likely the instability of carbon-bound S species in a hydrogen atmosphere at elevated temperatures.^70^ In anticipation of this instability, we annealed the CNF-S catalyst support at 450 °C in a H_2_ atmosphere before Co loading, thus deliberately exceeding the reduction temperature by 100 °C. Despite these efforts, the S species remained mobile, probably as a consequence of the presence of reactive atomic H via hydrogen spillover from metallic Co surfaces during reduction and FTS, transforming carbon-bound S into volatile catalyst poisons such as H_2_S. For spent Co/CNF-P, STEM–EDS mapping showed a further spatial redistribution of P species, concentrating around the location of Co nanoparticles (Figures 2a and S7). However, averaged EDS line scans of 5–10 Co NPs suggested a decline in P content of Co nanoparticles over 80 h TOS FTS, with P levels comparable to those of the pristine catalyst prior to Co_*x*_P_*y*_ formation during catalyst reduction (Figure 2b–d).

XPS of the spent catalysts compared well to the STEM–EDS and in situ magnetometry results, with the XPS Co 2p contribution indicating the absence of any Co_*x*_N_*y*_ species for Co/CNF-N after FTS (Figure S24). It should be noted that the N 1s region was strongly attenuated after FTS due to the coverage of high-molecular-weight hydrocarbons (as identified by the shape of the XPS C 1s contribution), which remained on the catalyst surface despite washing with hexane. Due to negligible FTS activity, hydrocarbon coverage was not a problem on Co/CNF-S after FTS. In this context, a shoulder in the Co 2p_2/3_ peak centered around 778.5 eV suggested the presence of cobalt sulfide species, thus supporting the evidence for substantial S poisoning (Figure 8d and S22). XPS of Co/CNF-P after FTS corroborated the STEM–EDS results as it was found that the cobalt phosphide (Co_*x*_P_*y*_) phase identified after catalyst reduction was mostly lost over 80 h FTS (Figure 8e and S24). Simultaneously, a shift of the P 2p contribution to higher binding energies was observed, with deconvolution of the P 2p region suggesting that the Co_*x*_P_*y*_ phase was oxidized to the more electron-deficient oxidic C_0–1_PO_3–4_ species during FTS (Figure S15). STEM–EDS mapping and line scans suggested that the resulting phosphorus oxides remained in spatial vicinity to the cobalt nanoparticles but did not increase the P concentration in the exact location of Co NPs. Considering the results of in situ magnetometry (Figure 8c), the Co fraction of the Co_*x*_P_*y*_ phase was likely converted to cobalt oxide species during FTS, meaning that the overall loss of Co^0^ observed by magnetometry was ultimately a consequence of cobalt reoxidation. Given the Co particle size (7.7 ± 1.7 nm after reduction), the strongly reducing conditions during FTS (e.g., at low CO conversion <20%),^5,32^ and the absence of reoxidation for the nondoped catalyst Co/CNF-H, cobalt reoxidation is most likely not an intrinsic consequence of the reaction conditions but is enabled by the presence of P.

## 4 Conclusion

Using a recently developed postsynthesis heteroatom doping method, N-, S-, and P-doped carbon nanofiber (CNF) supports were synthesized, each with similar heteroatom loading and comparable textural and nanostructural properties. These supports were then loaded with size-defined colloidal Co nanoparticles, yielding a series of highly defined Co-based FTS catalysts. The primary difference among these catalysts was the presence or absence of heteroatom dopants in the carbon support. Co nanoparticles on nondoped, N-doped, and S-doped CNFs were fully reducible, whereas Co on P-doped CNF exhibited much stronger metal–support interactions, resulting in a significantly lower degree of reduction. In situ characterization of the reduced Co/CNF catalysts revealed that heteroatom doping had a pronounced effect on the structure and properties of the catalytically active Co^0^ phase. This included changes in the Co coordination number, the crystal phase composition (fcc/hcp ratio), and the magnetic behavior.

Compared to the nondoped Co/CNF-H catalyst, the presence of N, S, or P proved detrimental to FTS activity and/or catalyst stability. Specifically, Co/CNF-N displayed high initial FTS activity but deactivated rapidly due to increased sintering and the loss of catalytically active Co^0^ through phase transformations that were absent in the nondoped reference catalyst. Co/CNF-S demonstrated favorable Co reducibility, but the mobility of carbon-bound sulfur species, most likely promoted by the hydrogen-rich atmosphere, led to severe sulfur poisoning and negligible FTS activity. Lastly, while strong metal–support interactions on Co/CNF-P helped to prevent deactivation by sintering, this came at the cost of low Co reducibility and the loss of active Co^0^ through the formation of cobalt phosphides and their subsequent decomposition into phosphorus oxides and cobalt oxides under FTS conditions.

These findings suggest that the benefits of carbon support heteroatom doping reported in previous studies may have been overestimated due to comparability issues, particularly the overlapping effects of Co particle size and support interactions. Our results challenge the existing literature and emphasize the detrimental impact of N, S, and P doping on the activity and/or stability of Co-based FTS catalysts, calling for a reassessment of design strategies of carbon-supported FTS catalysts.

## Supporting Information Available

Detailed fitting procedures for XPS, further characterization of the CNF supports: N_2_ physisorption isotherms, XRD patterns, Raman spectra, as well as XPS surveys and surface composition, and further characterization of Co/CNF catalysts: enlarged EDS elemental maps, further STEM images, Co particle size distributions, in situ XANES and EXAFS spectra including first shell fitting of the reduced Co/CNF catalysts, XRD including phase analysis by Rietveld refinement, XPS, in situ XANES-LCF analysis of catalyst reduction, and full-sized in situ magnetic hysteresis of the reduced Co/CNF catalysts (PDF)
