# Supplementary material for: Is Carbon Heteroatom Doping the Key to Active and Stable Carbon Supported Cobalt Fischer–Tropsch Catalysts?
Source: ACS Catal. 2025 Apr 9;15(8):6673–89. doi: 10.1021/acscatal.4c08092 (PMC12012828; doi:10.1021/acscatal.4c08092)
Supplement: Supplementary file 1 — cs4c08092_si_001.pdf [file cs4c08092_si_001.pdf]

Is carbon heteroatom doping the key to active and stable carbon supported cobalt Fischer-Tropsch catalysts?

Supplementary Information

Felix Herold[a,b,*], Dominic de Oliveira[c], Göran Baade[d], Jens Friedland[d], Robert Güttel[d], Michael Claeys[c], Magnus Rønning[a,*]

[a]Norwegian University of Science and Technology, Department of Chemical Engineering, Trondheim 7491, Norway

[b]Friedrich-Alexander-Universität Erlangen-Nürnberg, Institute for Power-to-X Technologies, 90762 Fürth, Germany

[c]University of Cape Town, Department of Chemical Engineering, Catalysis Institute, Rondebosch 7701, South Africa

[d]Ulm University, Institute of Chemical Engineering, 89081 Ulm, Germany

**Corresponding Authors*: F. Herold, felix.herold@ntnu.no; M. Rønning, magnus.ronning@ntnu.no

1. EXPERIMENTAL

X-ray photoelectron spectroscopy was performed on a Kratos Analytical Axis Ultra DLD spectrometer using monochromatic Al Kα irradiation (1486.6 eV) operating the anode at 10 kV with an aperture of 700x300 μm. The energy axis was calibrated by fixing the C 1s contribution of sp^2 carbon ("graphite") at 284.6 eV. For deconvolution, linear combinations of Gaussian and Lorentzian functions were utilized (pseudo-Voigt-profiles) and Shirley background subtraction was performed prior to fitting.

Deconvolution of the N 1s envelope was performed assuming the contribution of pyridinic nitrogen at 398.3 ± 0.2 eV, that of pyrrolic nitrogen at 399.7 ± 0.2 eV, that of quaternary nitrogen species at 401.0 ± 0.2 eV and that of oxidized N at 403.4 ± 0.2 eV.[1,2] The full width at half maximum (FWHM) was restricted to 1.5 ± 0.1 eV. Shape factors (ratio of Lorentzian/Gaussian functions) were kept equal for all functions during the fit and were usually close to 0.5.

The S 2p contribution was deconvoluted with doublets of two pseudo-Voigt profiles with fixed intensity ratio (0.55 ± 0.05) and fixed relative binding energies (Δ = 1.18 eV) to represent the $2p_{1/2}$ and $2p_{3/2}$ contribution of each sulfur species. The following binding energies relate to the $2p_{3/2}$ contribution of each S species: the binding energy of aliphatic sulfides was assumed to be located at 162 ± 0.3 eV, that of aromatic sulfides (thiophenes) at 164.0 ± 0.2 eV and that of oxidized sulfur species (sulfones, sulfoxides) at 167.0 ± 0.75 eV.[3,4] The full width at half maximum (FWHM) was restricted to values of 1.2 ± 0.2 eV. Shape factors (ratio of Lorentzian/Gaussian functions) were kept equal for all functions during the fit and were usually close to 0.5.

The P 2p contribution was deconvoluted with doublets of two pseudo-Voigt profiles with fixed intensity ratio (0.65±0.05) and fixed relative binding energies (Δ = 0.84 eV) to represent the

$2p_{1/2}$ and $2p_{3/2}$ contribution of each phosphorus species. The following binding energies relate to the $2p_{3/2}$ contribution of each P species: the binding energy of reduced phosphorus corresponding to C_3PO_1 (e. g. triphenylphosphin oxide as model compound)[5] was assumed to be located at 132.8 ± 0.1 eV, while the binding energy of oxidized phosphorus corresponding to $C_{0-1}PO_{3-4}$ (e. g. phosphonates) was assumed to be situated at 134.0 ± 0.1 eV.[3,6] The full width at half maximum (FWHM) was restricted to 1.5±0.1 eV. Shape factors (ratio of Lorentzian/Gaussian functions) were kept equal for all functions during the fit and were usually close to 0.5.

2. RESULTS

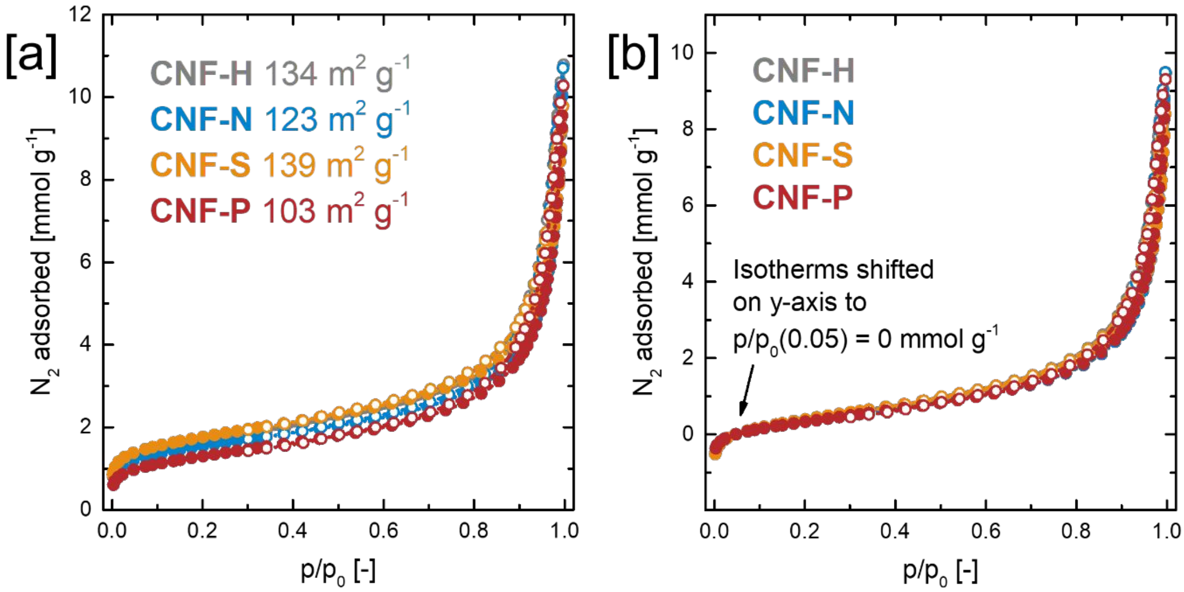

Figure S1. [a] N_2 physisorption isotherms of CNF supports, including BET specific surface areas. [b] N_2 physisorption isotherms of CNF supports shifted on the y-axis to $p/p_0(0.05)$ = 0 mmol g^{-1}.

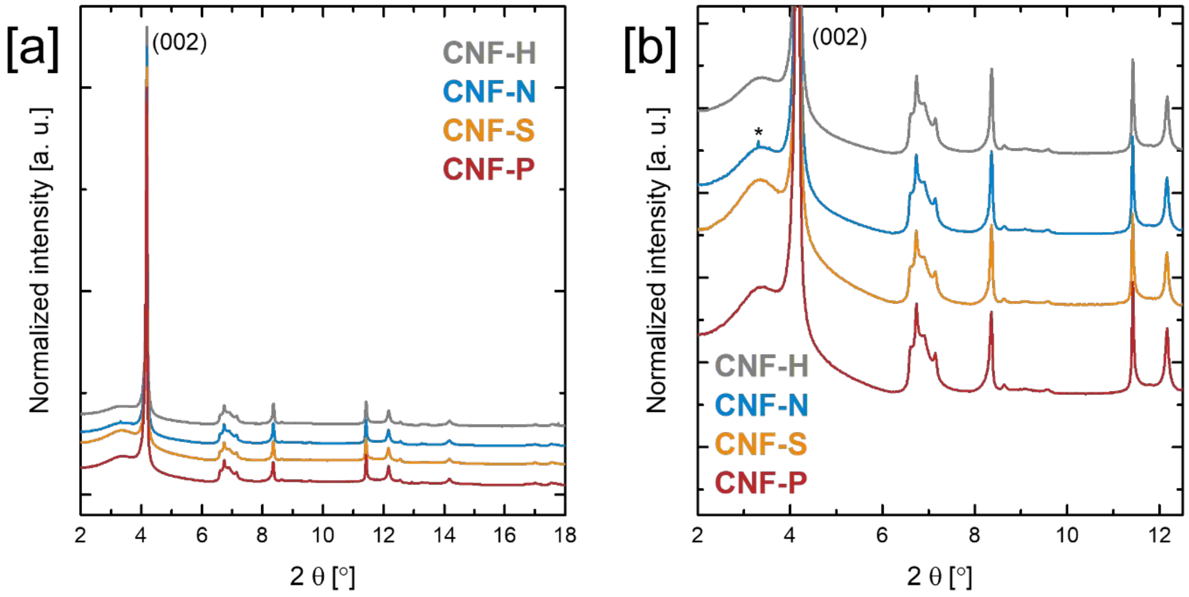

Figure S2. X-ray diffraction patterns of the CNF supports. [a] Full patterns and [b] enlarged display of the region between 2 and 12.5 ° / 2θ. It should be noted that deviations in the broad signal centred around 3.5 ° / 2θ stem from the quartz capillaries that were used as sample holders at the beamline. The asterisk marks an artifact of the measurement.

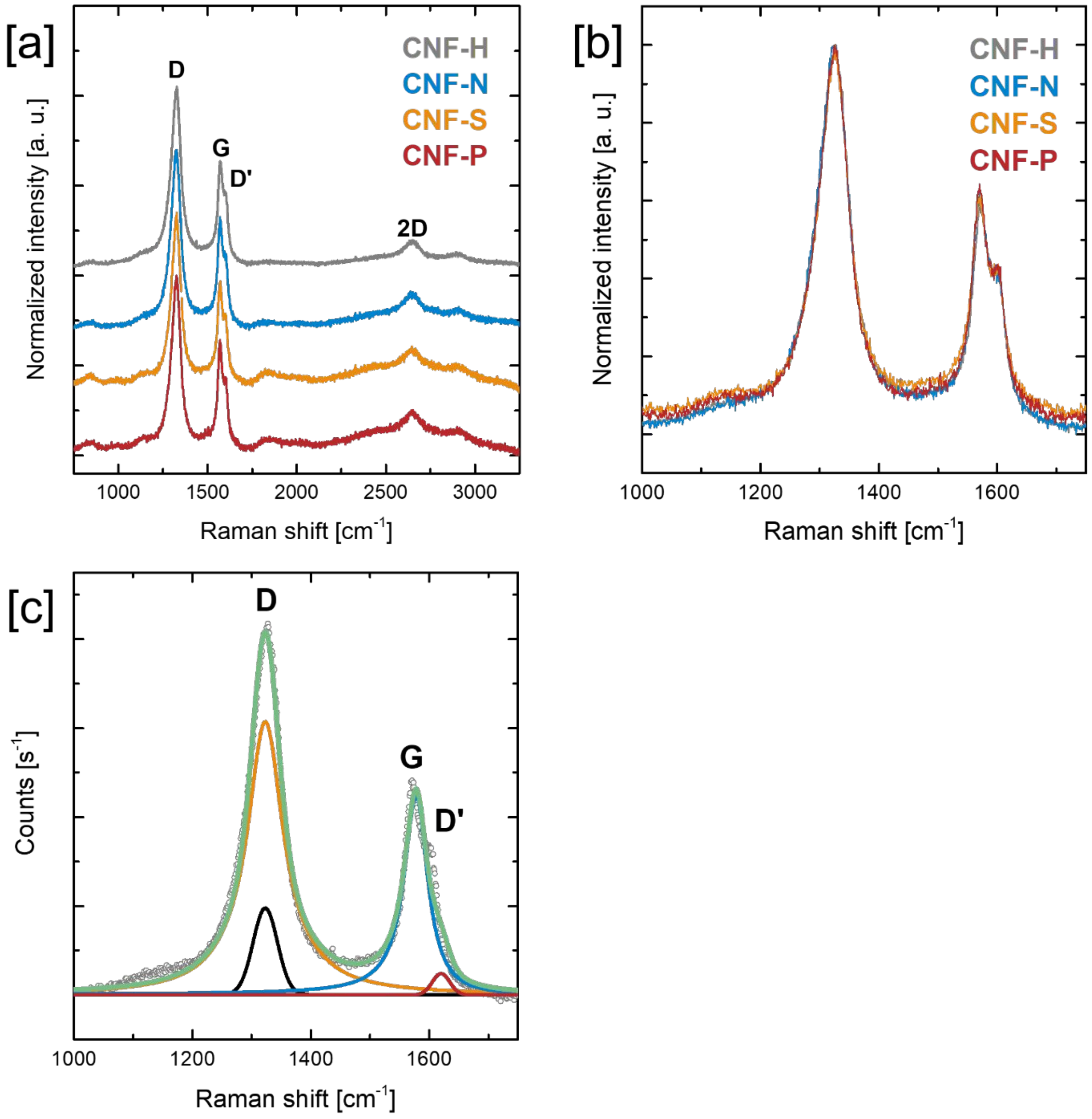

Figure S3. [a] Representative Raman spectra of the CNF supports [b] overlay of representative Raman spectra between 100 and 1750 cm^{-1} and [c] D- and G-band deconvolution for CNF-H according to a procedure proposed by Mallet-Ladeira *et al.*[7]

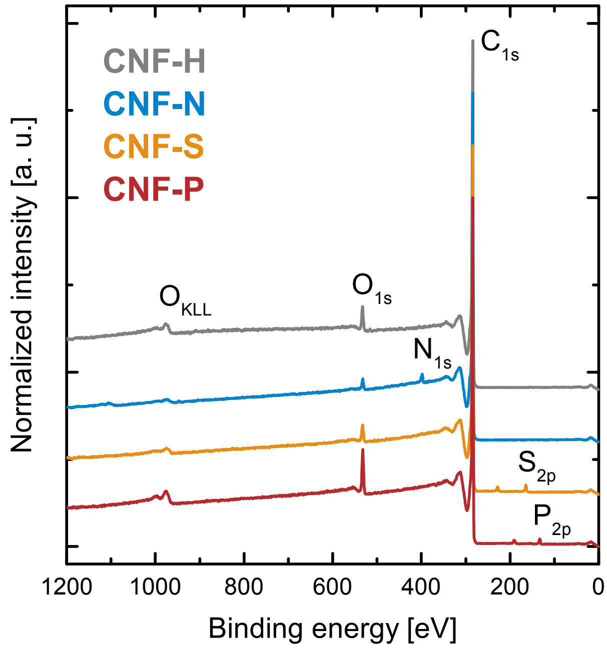

Figure S4. XPS survey spectra of the CNF supports.

Table S1. XPS surface composition of the pristine catalyst supports.

Sample	C [at.%]	O [at.%]	N [at.%]	S [at.%]	P [at.%]
**CNF-H**	97.7	2.3	-	-	-
**CNF-N**	97.5	1.1	1.4	-	-
**CNF-S**	96.8	1.8	-	1.4	-
**CNF-P**	94.5	4.0	-	-	1.5

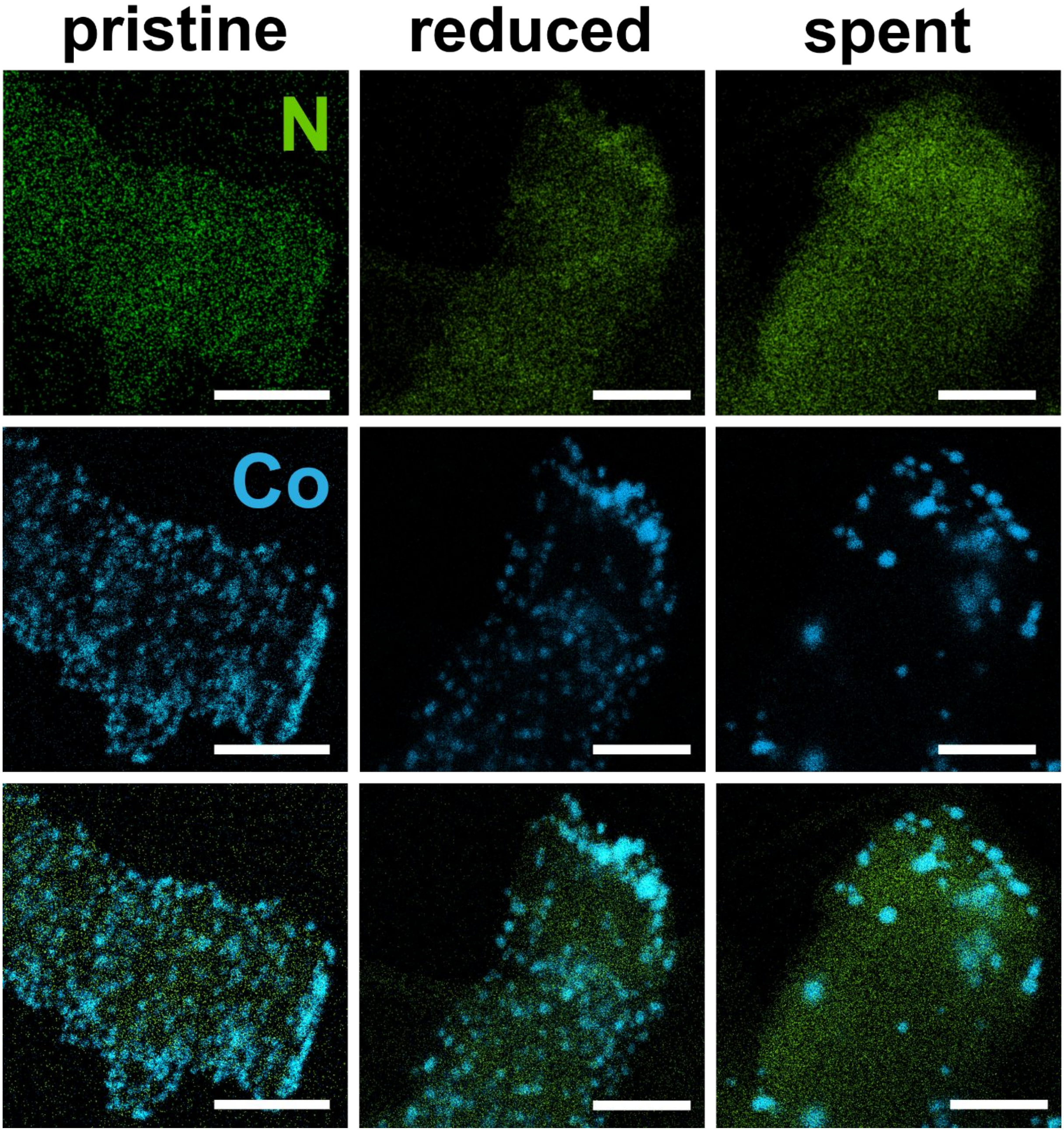

Figure S5. EDS elemental maps of pristine, reduced/passivated and spent/passivated (after 80 h FTS) Co/CNF-N catalysts. The scale bars indicate 100 nm.

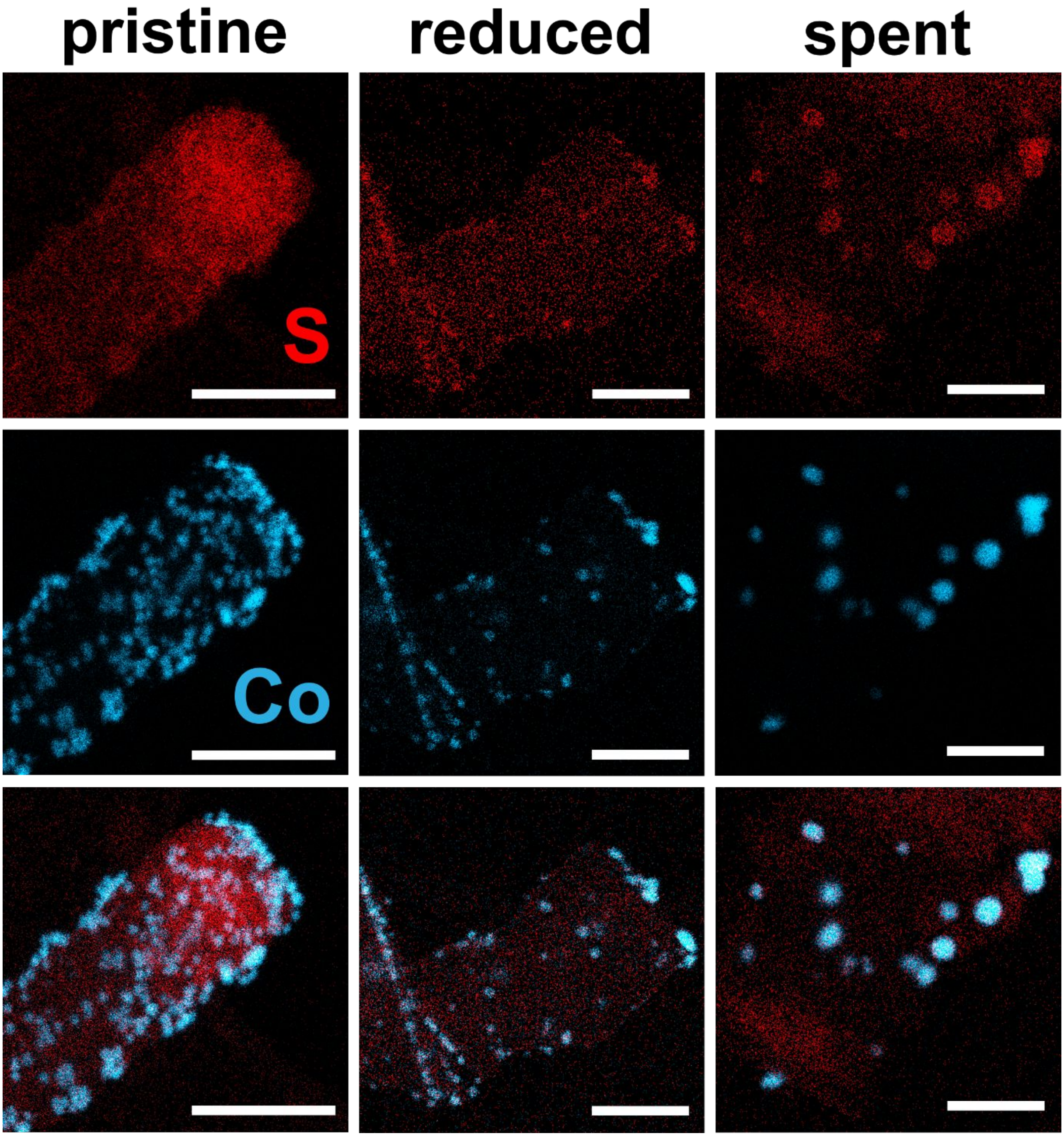

Figure S6. EDS elemental maps of pristine, reduced/passivated and spent/passivated (after 80 h FTS) Co/CNF-S catalysts. The scale bars indicate 100 nm.

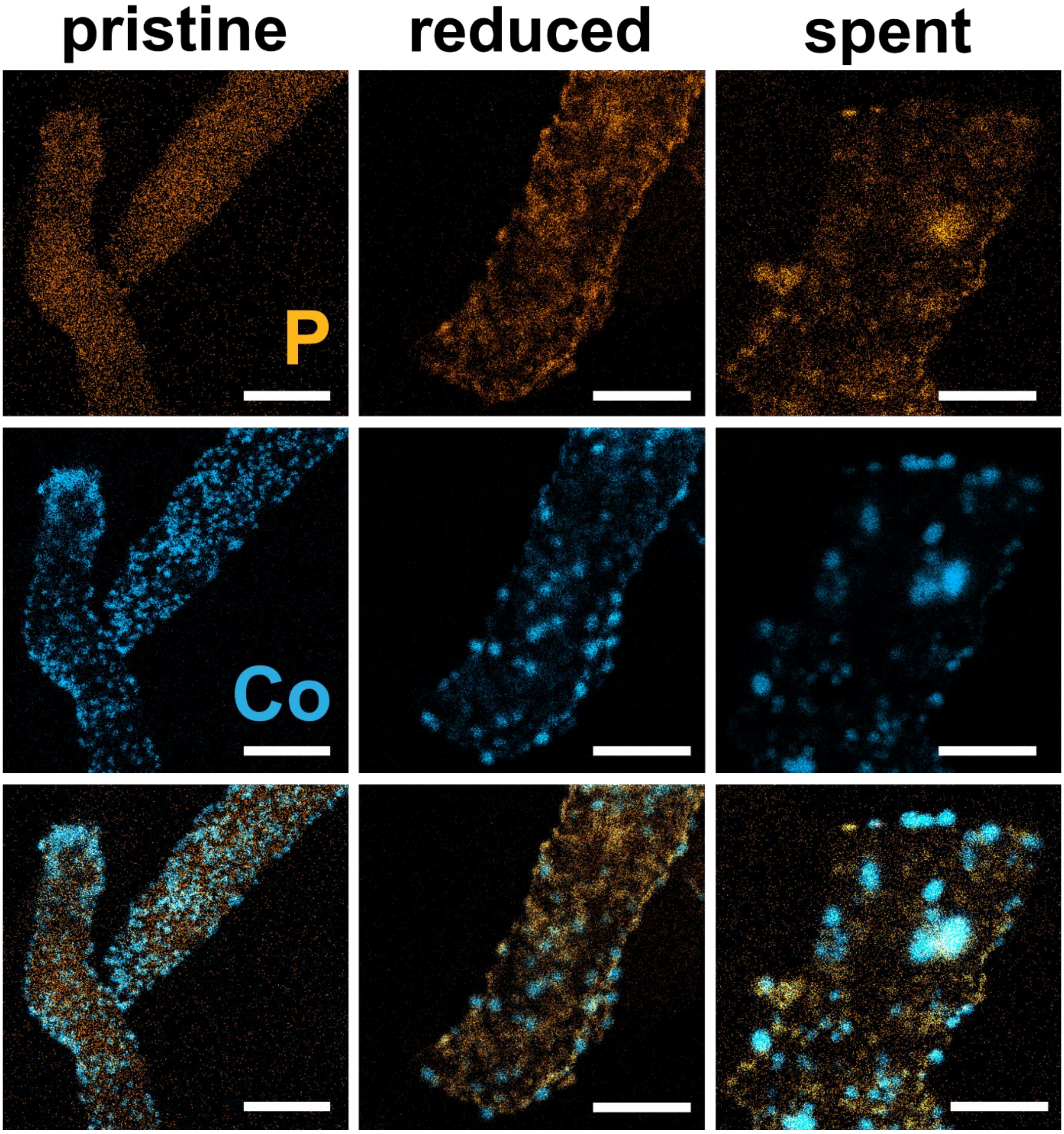

Figure S7. EDS elemental maps of pristine, reduced/passivated and spent/passivated (after 80 h FTS) Co/CNF-P catalysts. The scale bars indicate 100 nm.

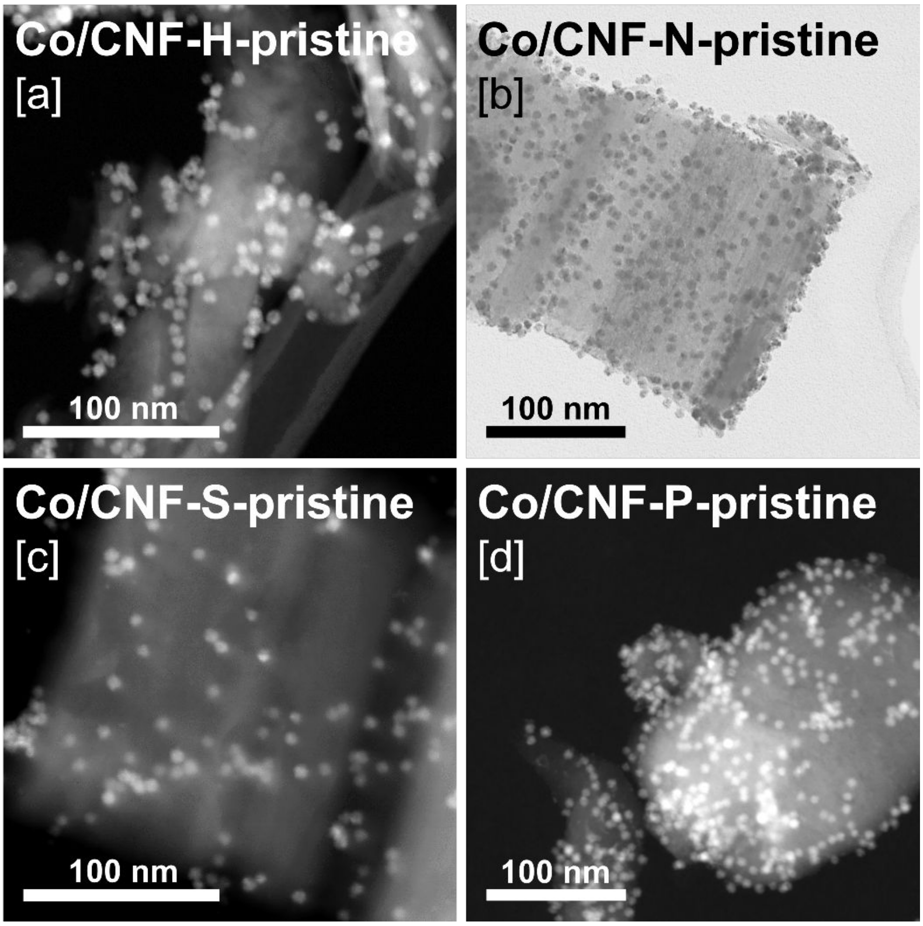

Figure S8. STEM micrographs of pristine Co/CNF catalysts. [a] HAADF image of Co/CNF-H, [b] brightfield image of Co/CNF-N, [c] HAADF image of Co/CNF-S and [d] HAADF image of Co/CNF-P.

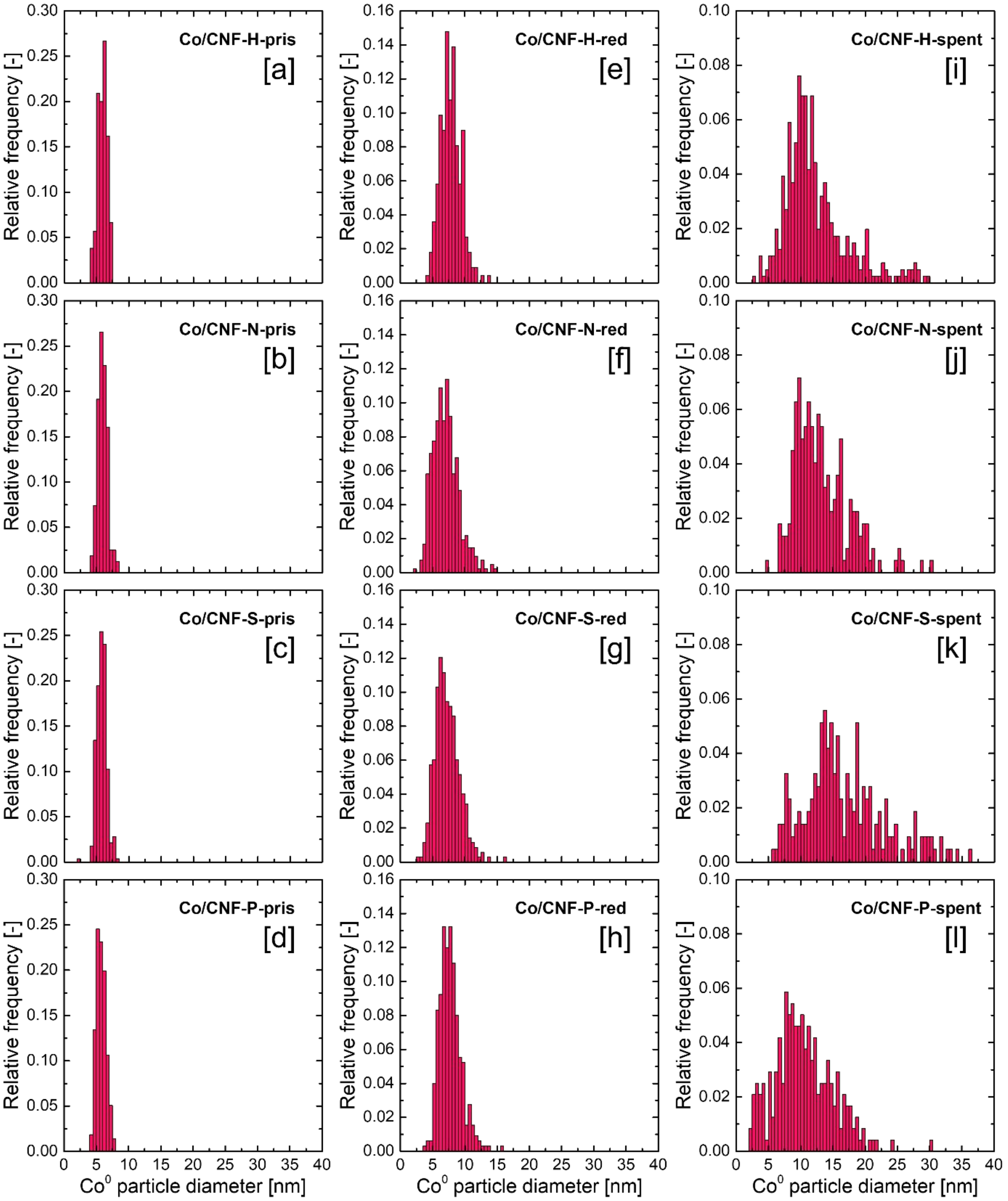

Figure S9. Co^0 particle size distributions determined from STEM images. Co particle size distributions of the [a-d] pristine Co/CNF catalysts, [e-h] reduced/passivated Co/CNF catalysts and [i-l] spent/passivated (after 80 h FTS) Co/CNF catalysts.

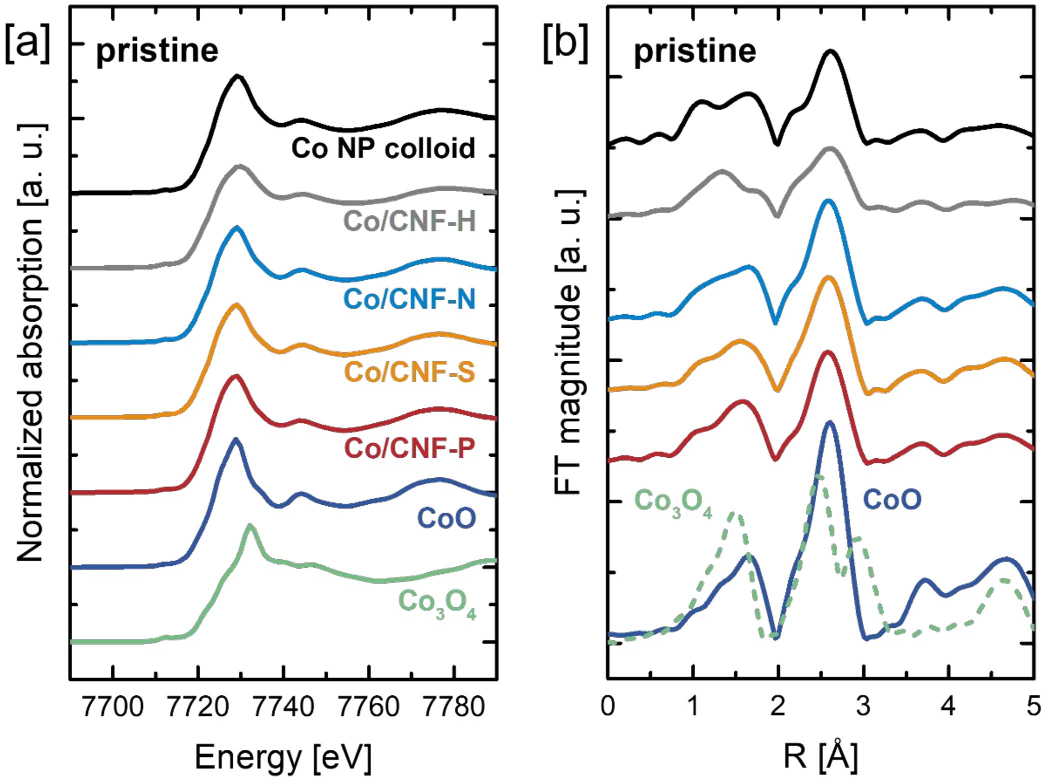

Figure S10. [a] Co K-edge XANES spectra and [b] k^2 weighted EXAFS spectra in R space of the Co nanoparticle colloid, the pristine Co/CNF catalysts as well as CoO and Co_3O_4 standards.

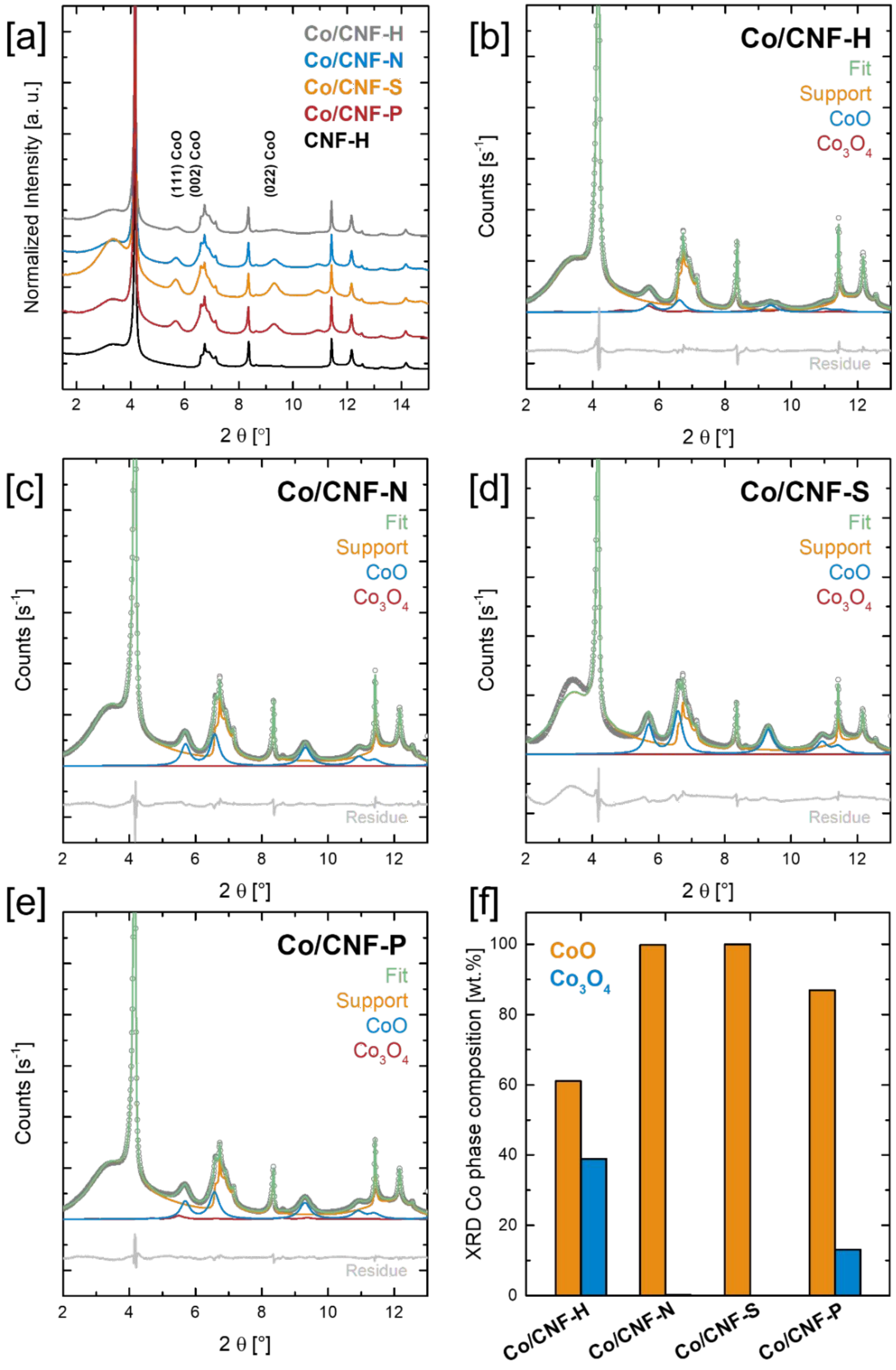

Figure S11. [a] XRD patterns of the pristine Co/CNF catalysts. Rietveld refinement of XRD patterns of pristine [b] Co/CNF-H, [c] Co/CNF-N, [d] Co/CNF-S and [e] Co/CNF-P. [f] Co phase composition of the pristine Co/CNF catalysts as determined by Rietveld refinement of the XRD patterns.

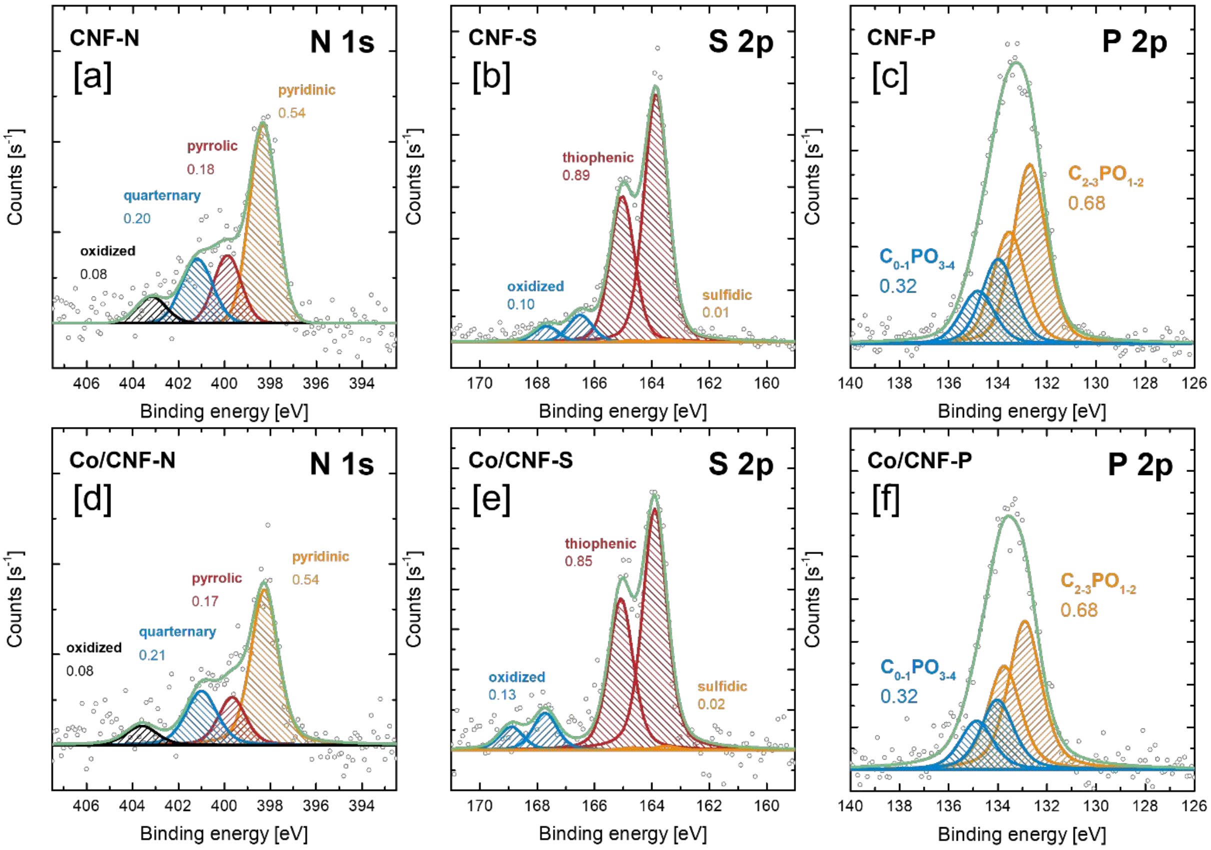

Figure S12. Deconvolution of high resolution XPS spectra of the pristine CNF supports and the pristine Co/CNF catalysts. [a] N 1s contribution of CNF-N, [b] S 2p contribution of CNF-S and [c] P 2p contribution. [d] N 1s contribution of pristine Co/CNF-N, [e] S 2p contribution of pristine Co/CNF-S and [f] P 2p contribution of Co/CNF-P.

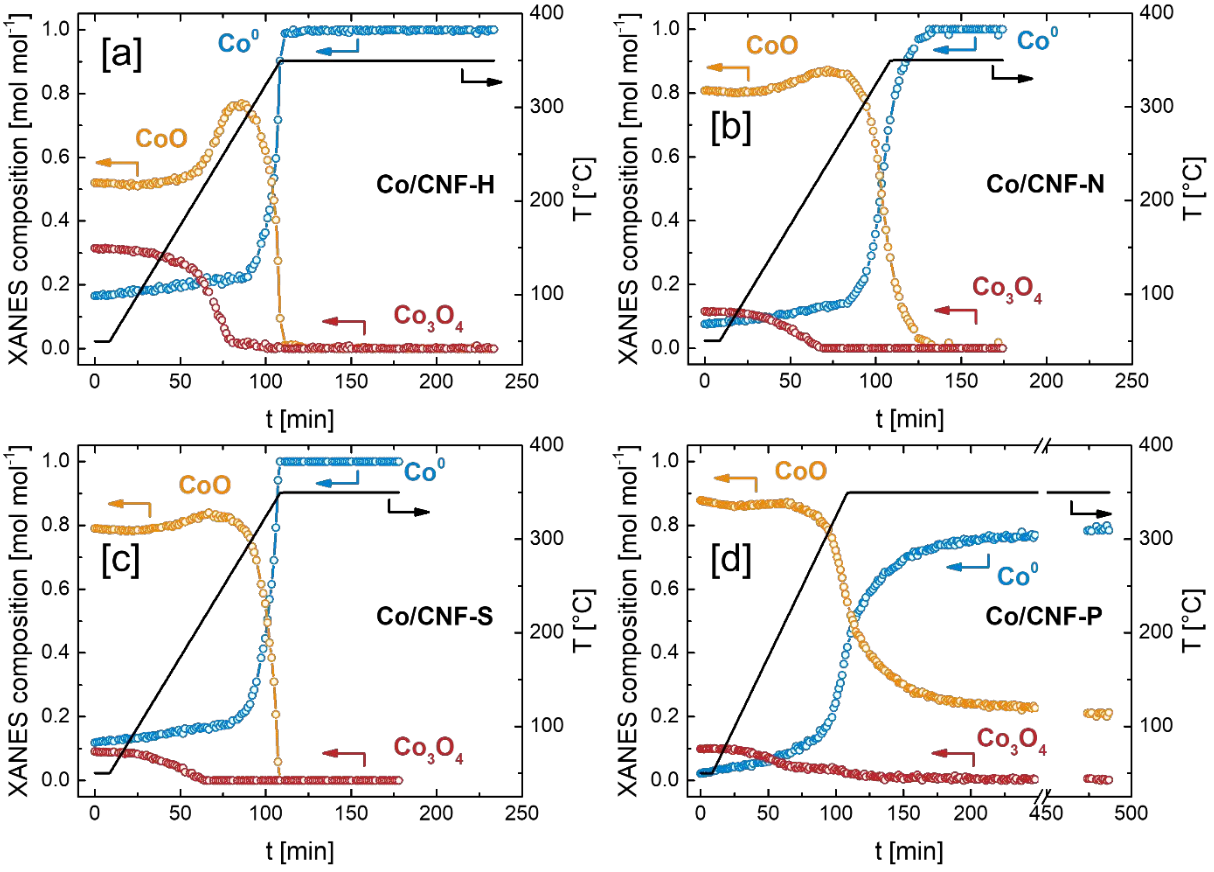

Figure S13. Results of *in situ* Co K-edge XANES LCF analysis of the catalyst reduction (3 °C min^{-1} to 350 °C, 25 vol.% H_2 in He). Composition of Co species of [a] Co/CNF-H, [b] Co/CNF-N, [c] Co/CNF-S and [d] Co/CNF-P.

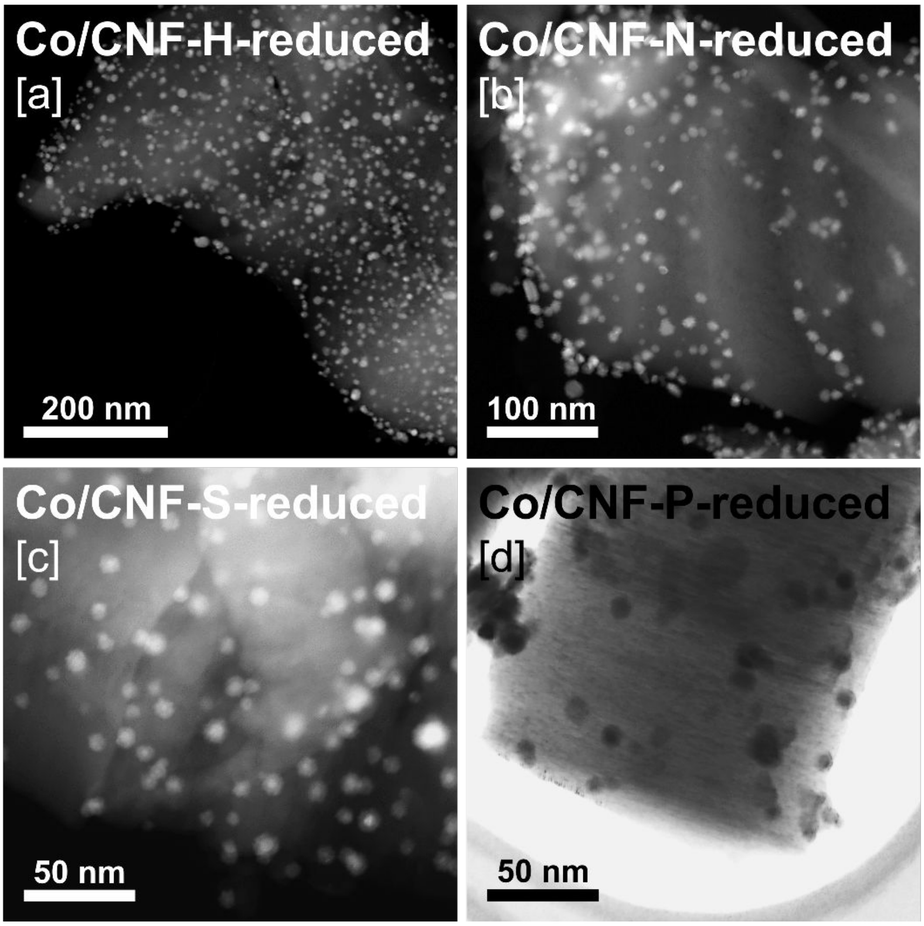

Figure S14. STEM micrographs of reduced and passivated Co/CNF catalysts. [a] HAADF image of Co/CNF-H, [b] HAADF image of Co/CNF-N, [c] HAADF image of Co/CNF-S and [d] brightfield image of Co/CNF-P.

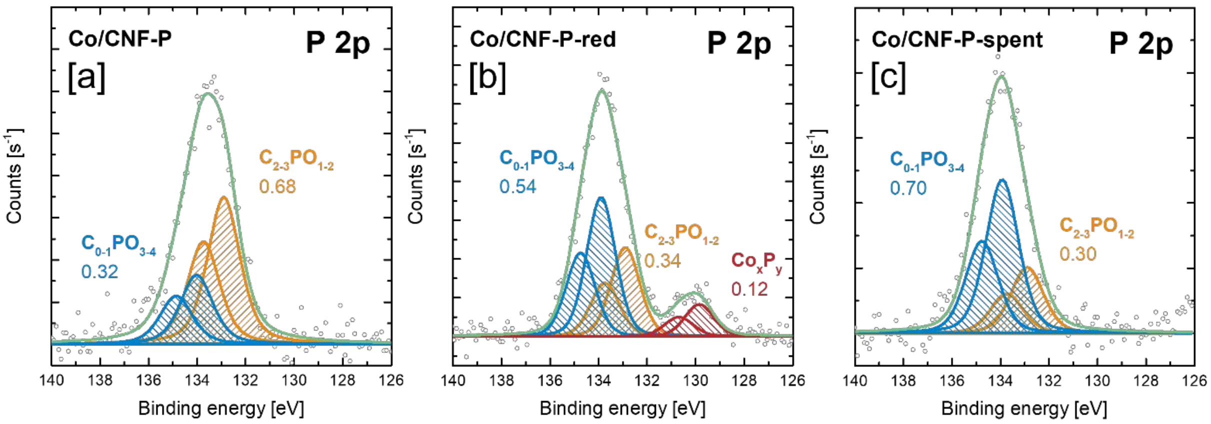

Figure S15.Deconvolution of the XPS P 2p contribution of [a] pristine Co/CNF-P, [b] reduced/passivated Co/CNF-P and [c] spent/passivated (after 80 h FTS) Co/CNF-P.

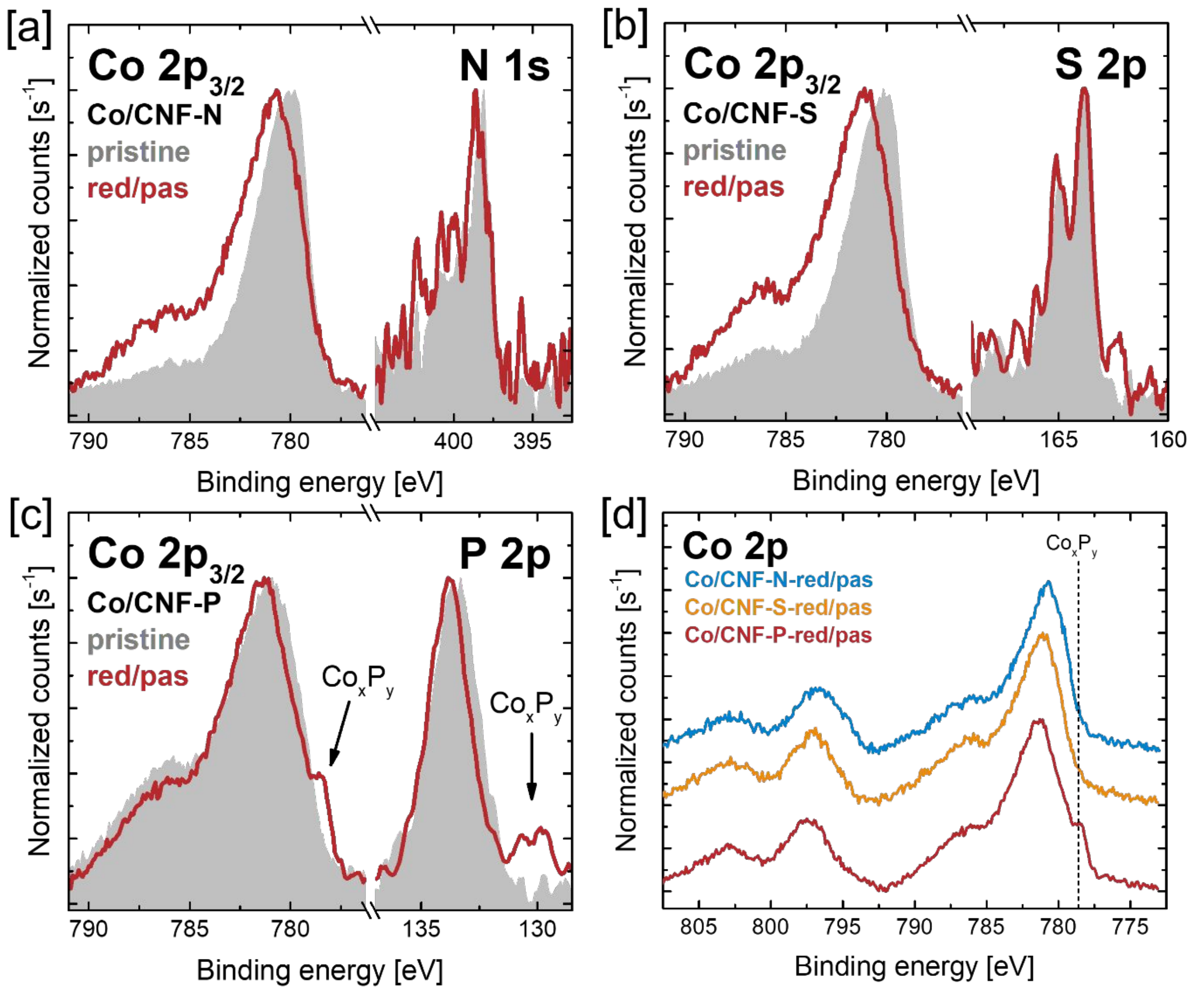

Figure S16. Comparison of XPS spectra of the Co/CNF catalysts before and after reduction. [a] Comparison of the XPS N 1s and Co $2p_{3/2}$ regions before and after reduction/passivation of Co/CNF-N. [b] Comparison of the XPS S 2p and Co $2p_{3/2}$ regions before and after reduction/passivation of Co/CNF-S. [c] Comparison of the XPS P 2p and Co $2p_{3/2}$ regions before and after reduction/passivation of Co/CNF-P. [d] Normalized Co 2p contributions of reduced/passivated Co/CNF-N, Co/CNF-S and Co/CNF-P.

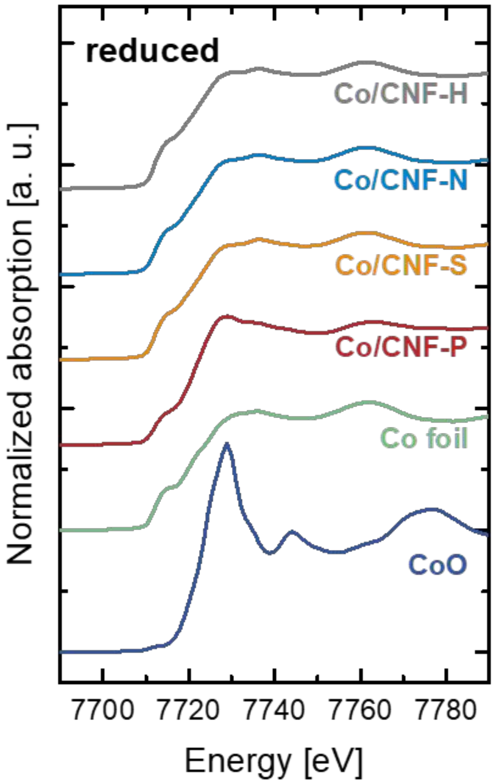

Figure S17. *In situ* Co K-edge XANES spectra of the reduced Co/CNF catalysts as well as Co and CoO standards.

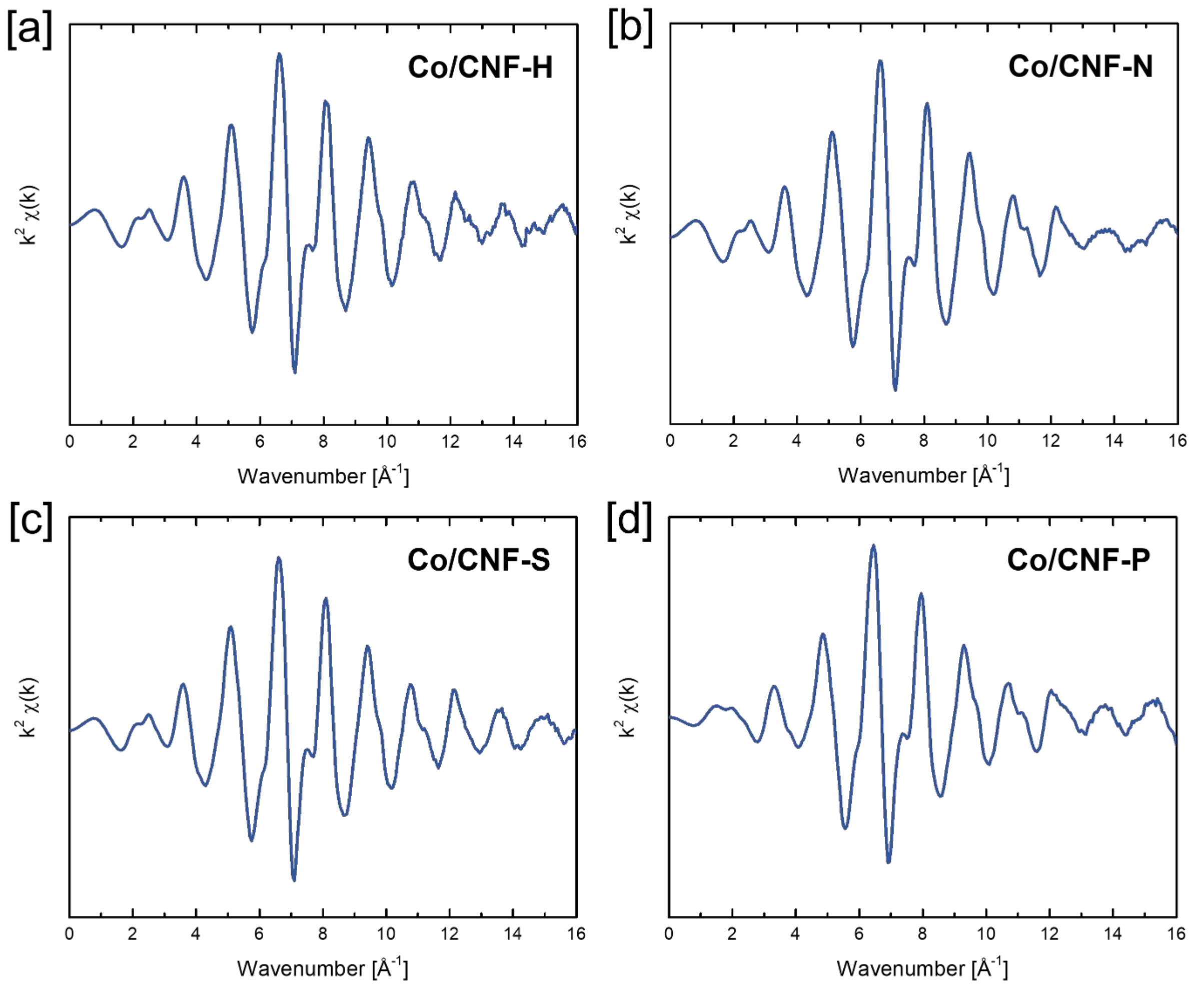

Figure S18. k^2 weighted *in situ* EXAFS spectra of reduced [a] Co/CNF-H, [b] Co/CNF-N, [c] Co/CNF-S, [d] Co/CNF-P.

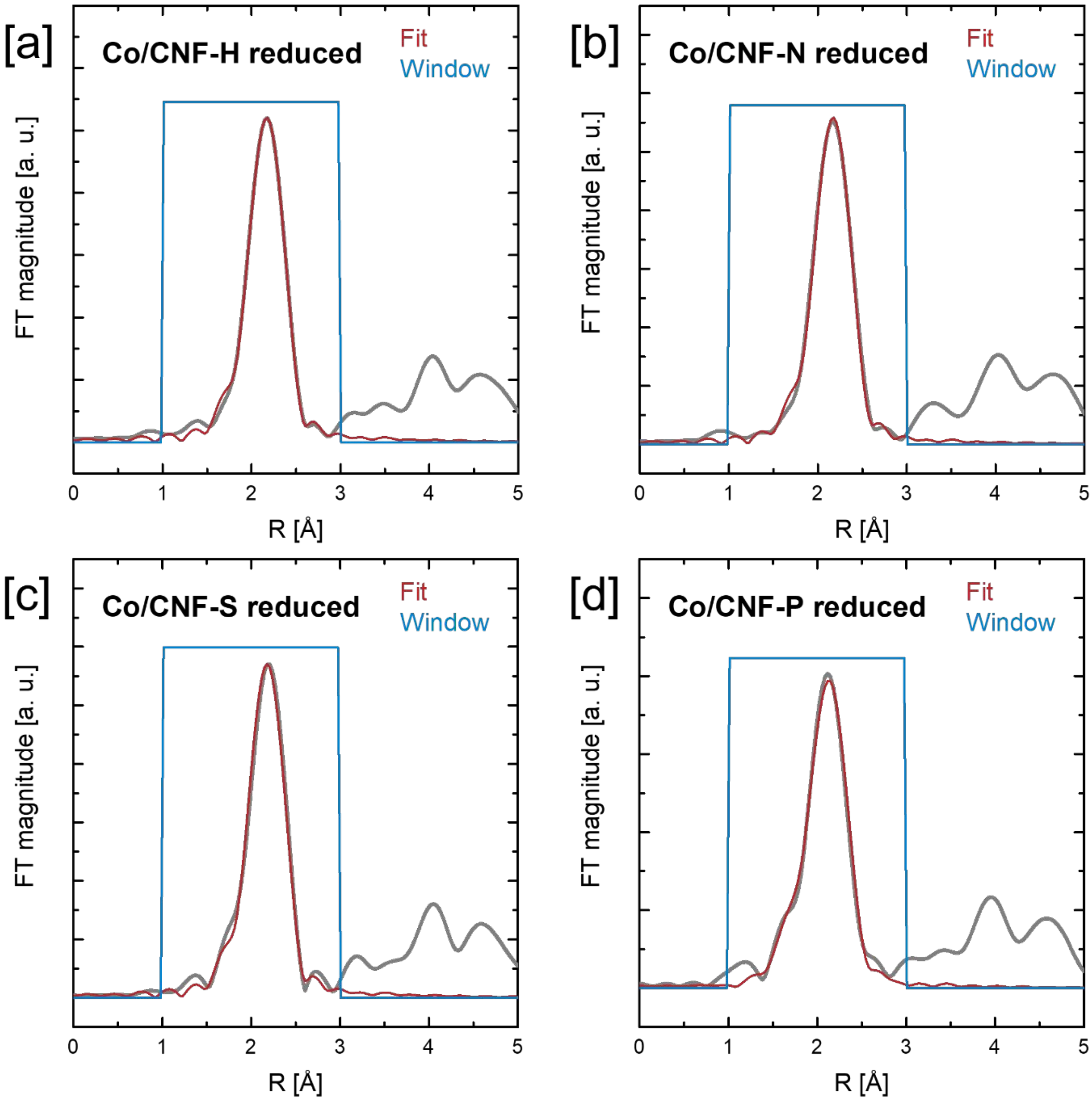

Figure S19. First shell fitting of k^2 weighted *in situ* EXAFS data in R space of reduced [a] Co/CNF-H, [b] Co/CNF-N, [c] Co/CNF-S, [d] Co/CNF-P.

Table S2. First-shell fitting parameters of *in situ* EXAFS data measured after catalyst reduction.

Sample	Phase	Scattering path	E_0 [eV]	CN	R [Å]	σ^2 [Å]	R-factor
**Co/CNF-H**	Co^0	Co-Co	5.69	8.7	2.484	0.006	0.004
**Co/CNF-N**	Co^0	Co-Co	6.12	10.0	2.484	0.007	0.004
**Co/CNF-S**	Co^0	Co-Co	6.02	9.4	2.489	0.006	0.006
	Co^0	Co-Co		11.0	2.482	0.008	
**Co/CNF-P**	CoO	Co-O	-3.87	5.5	2.02	0.008	0.010
	CoO	Co-Co		11.0	2.86	0.023	

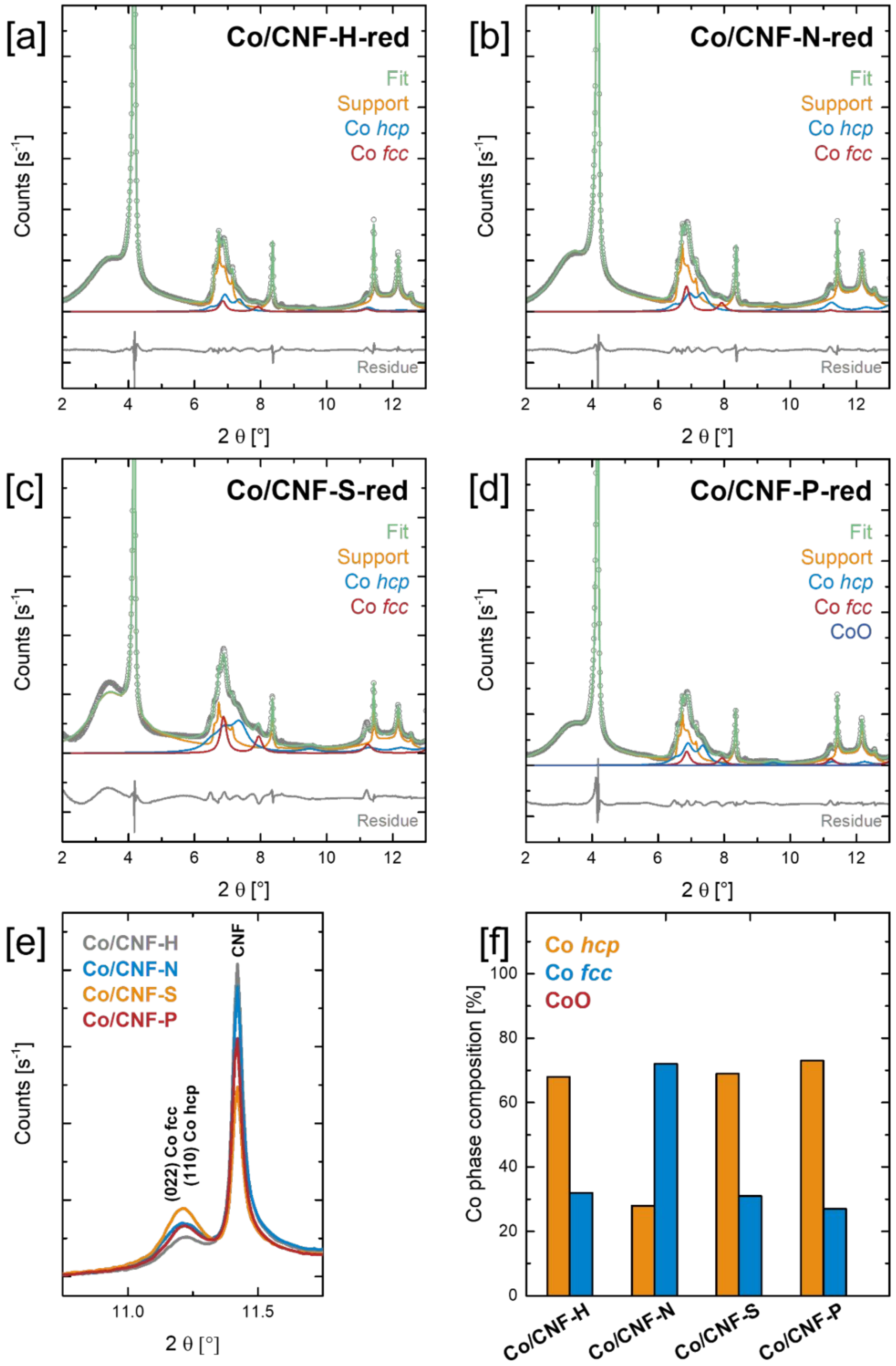

Figure S20. Rietveld refinement of *in situ* XRD patterns of reduced [a] Co/CNF-H, [b] Co/CNF-N, [c] Co/CNF-S and [d] Co/CNF-P. [e] Co hcp(110)/fcc(022) reflex, that is used to compare the relative Co crystallite size by analysis of the FWHM. [f] Co phase composition of the reduced Co/CNF catalysts as determined by Rietveld refinement.

Table S3. Rietveld refinement of *in situ* XRD patterns after catalyst reduction.

Sample	Co *hcp* [wt.%]	Co *fcc* [wt.%]	CoO [wt.%]	Co *hcp* crystallite size [nm]	Co *fcc* crystallite size [nm]	FWHM Co hcp (110) / fcc (022) [°]
**Co/CNF-H**	68	32	-	4.4	6.9	0.15
**Co/CNF-N**	28	72	-	3.4	10.1	0.16
**Co/CNF-S**	69	31	-	1.9	7.7	0.16
**Co/CNF-P**	73	27	0	3.7	6.8	0.16

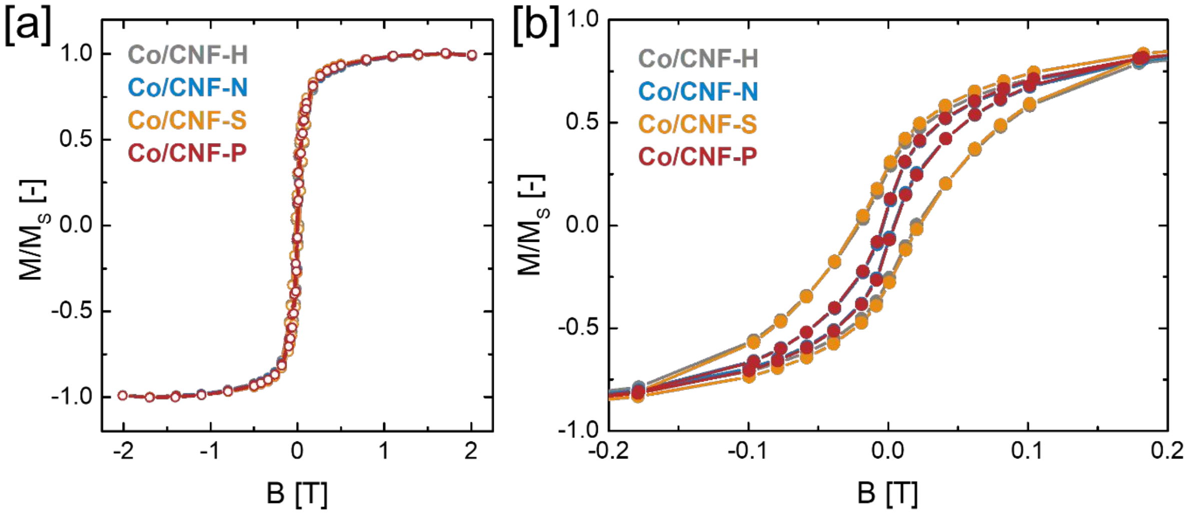

Figure S21. Magnetic hysteresis measured *in situ* at 350 °C in 25 vol.% H_2 in He of the reduced Co/CNF catalysts. [a] Full sized hysteresis from -2 to 2 T, [b] enlarged view of the hysteresis between -0.2 and 0.2 T.

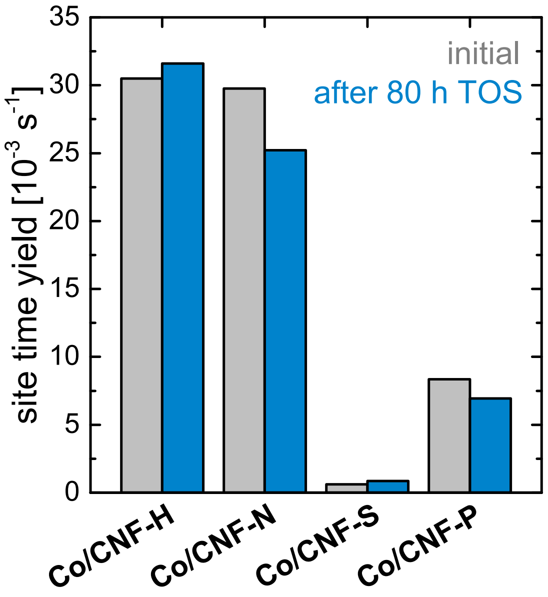

Figure S22. Initial and final site time yields of all catalysts. STY's were determined with the STEM mean particle sizes of the reduced and spent catalysts, assuming spherical Co nanoparticles.

Table S4. FTS selectivity data after catalyst induction (~17 h TOS).

**Sample**	**$X_{CO, ai}$ [%]**	**CTY_{ai}***	**$S_{CH4, ai}$ [%$_C$]**	**$S_{C2-C4, ai}$ [%$_C$]**	**$S_{C5+, ai}$ [%$_C$]**	**$O/P_{C3, ai}$# [%$_C$]**	**α_{ai}§**
**Co/CNF-H**	24	4.5	21	13	66	1.2	0.71
**Co/CNF-N**	17	2.9	27	13	60	1.1	0.72
**Co/CNF-S**	1	0.1	-	-	-	-	-
**Co/CNF-P**	20	1.5	29	17	54	0.4	0.68

$_{ai}$ after induction, reported at comparable conversion after the initiation period at ~17 h TOS.

*Cobalt time yield, reported as 10^{-5} mol_{CO} g_{Co} s^{-1}.

#Molar olefin to paraffin ratio of the C_3 products.

§Determined from the $C_1 – C_6$ products.

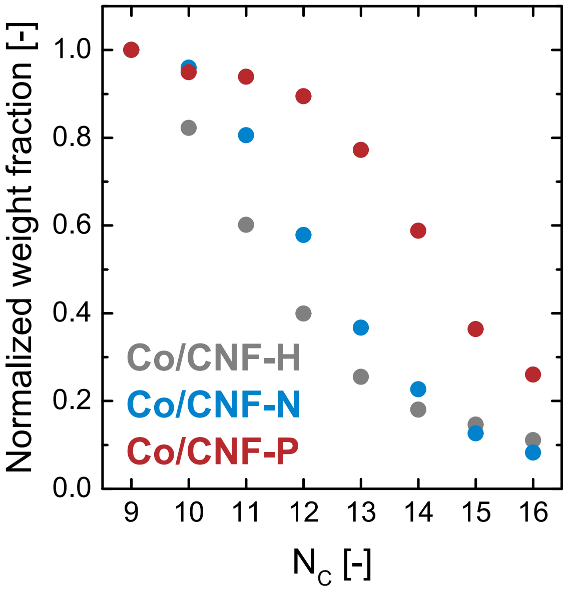

Figure S23. Relative weight fraction of the $C_9 – C_{16}$ products of Co/CNF-H, Co/CNF-N and Co/CNF-P, normalized to C_9 and sampled after 80 h TOS.

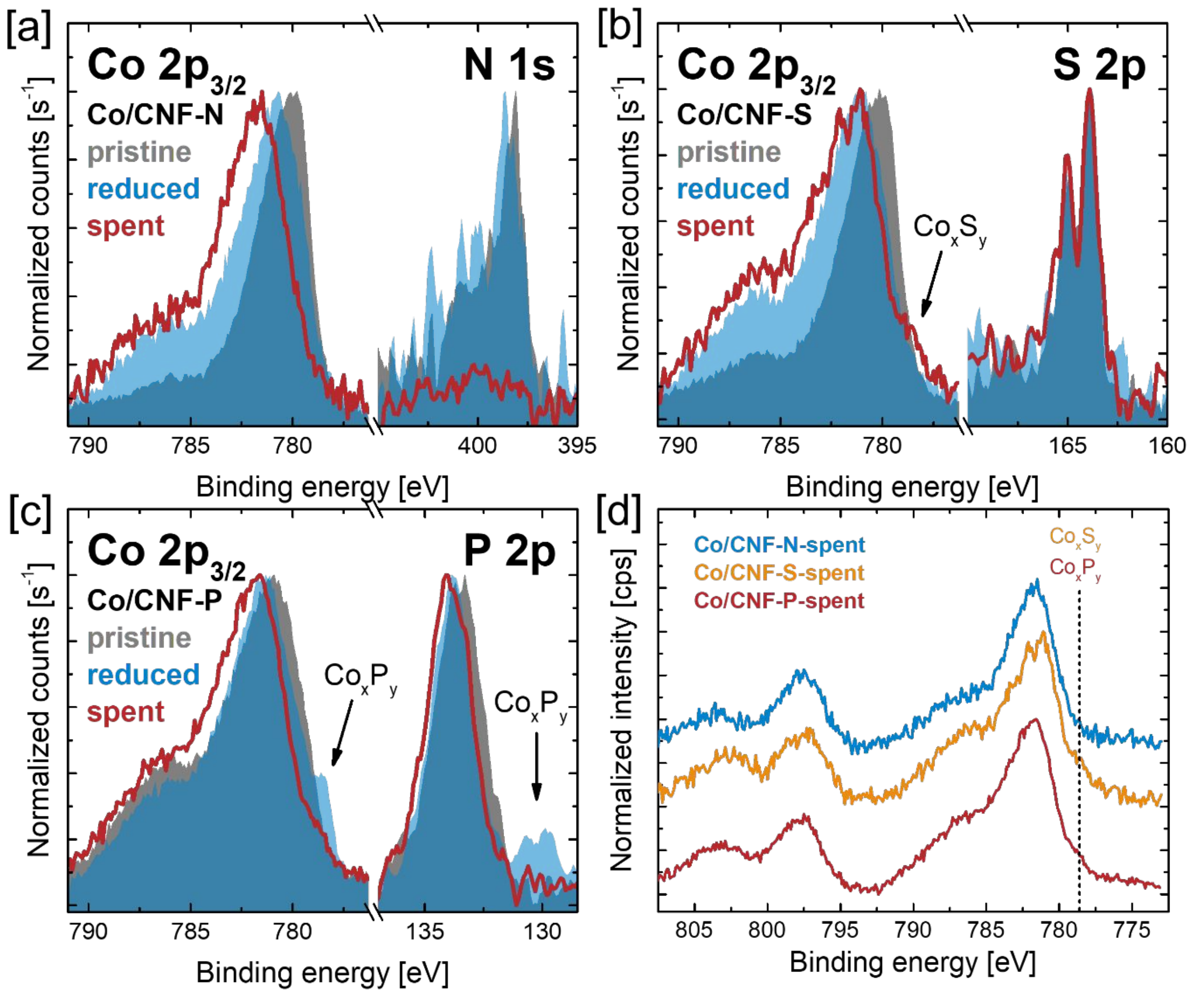

Figure S24. Comparison of XPS spectra of the pristine, reduced/passivated and spent/passivated (after 80 h FTS) Co/CNF catalysts [a] Comparison of the XPS N 1s and Co $2p_{3/2}$ regions of Co/CNF-N. [b] Comparison of the XPS S 2p and Co $2p_{3/2}$ regions of Co/CNF-S. [c] Comparison of the XPS P 2p and Co $2p_{3/2}$ regions of Co/CNF-P. [d] Normalized Co 2p contributions of spent/passivated Co/CNF-N, Co/CNF-S and Co/CNF-P.

REFERENCES

[1] X. Cui, Z. Pan, L. Zhang, H. Peng, G. Zheng, Selective Etching of Nitrogen-Doped Carbon by Steam for Enhanced Electrochemical CO 2 Reduction, Adv. Energy Mater. 7 (2017) 1701456. https://doi.org/10.1002/aenm.201701456.

[2] K. Friedel Ortega, R. Arrigo, B. Frank, R. Schlögl, A. Trunschke, Acid–Base Properties of N-Doped Carbon Nanotubes, Chem. Mater. 28 (2016) 6826–6839. https://doi.org/10.1021/acs.chemmater.6b01594.

[3] J.F. Moulder, W.F. Stickle, P.E. Sobol, K.D. Bomben, Handbook of X-ray Photoelectron Spectroscopy: A Reference Book of Standard Spectra for Identification and Interpretation of XPS Data, Perkin-Elmer Corporation, Eden Prairie, Minnesota, US, 1993.

[4] A.P. Terzyk, The influence of activated carbon surface chemical composition on the adsorption of acetaminophen (paracetamol) in vitro, Colloids Surf., A 177 (2001) 23–45. https://doi.org/10.1016/S0927-7757(00)00594-X.

[5] W.E. Morgan, W.J. Stec, R.G. Albridge, J.R. van Wazer, pi.-Bond feedback interpreted from the binding energy of the “2p” electrons of phosphorus, Inorg. Chem. 10 (1971) 926–930. https://doi.org/10.1021/ic50099a013.

[6] J. Wu, C. Jin, Z. Yang, J. Tian, R. Yang, Synthesis of phosphorus-doped carbon hollow spheres as efficient metal-free electrocatalysts for oxygen reduction, Carbon 82 (2015) 562–571. https://doi.org/10.1016/j.carbon.2014.11.008.

[7] P. Mallet-Ladeira, P. Puech, C. Toulouse, M. Cazayous, N. Ratel-Ramond, P. Weisbecker, G.L. Vignoles, M. Monthioux, A Raman study to obtain crystallite size of carbon materials, Carbon 80 (2014) 629–639. https://doi.org/10.1016/j.carbon.2014.09.006.
